# Trophic Plasticity of the Invasive Redbelly Tilapia (*Coptodon zillii*) in China Inferred From DNA Metabarcoding Analysis

**DOI:** 10.1002/ece3.71118

**Published:** 2025-04-03

**Authors:** Shoujie Tang, Ying Xing, Temesgen Tola Geletu, Jinliang Zhao

**Affiliations:** ^1^ Key Laboratory of Freshwater Aquatic Genetic Resources, Ministry of Agriculture and Rural Affairs Shanghai Ocean University Shanghai China; ^2^ Shanghai Collaborative Innovation for Aquatic Animal Genetics and Breeding Shanghai Ocean University Shanghai China; ^3^ National Demonstration Center for Experimental Fisheries Science Education Shanghai Ocean University Shanghai China; ^4^ School of Biological Sciences and Biotechnology Haramaya University Dire Dawa Ethiopia

**Keywords:** *Coptodon zillii*, DNA metabarcoding, feeding strategy, invasive species, trophic plasticity

## Abstract

The redbelly tilapia (*Coptodon zillii*) is one of the most dangerous invasive alien fishes in the world. In order to better understand the feeding patterns of invasive populations in different habitats and seasons, and to reveal the possible force of differences in dietary composition among populations, we used DNA metabarcoding technology to analyze the dietary composition of 23 specimens from five different water bodies (two rivers and three reservoirs) in southern China, and 60 specimens from Shuikou Reservoir in four seasons (spring, summer, fall, and winter). The results showed that samples from five different water bodies and four seasons in Shuikou Reservoir were annotated to a total of 22 and 37 phyla of food categories, respectively. Generalist trophic strategies were dominant in *C. zillii* populations. There was significant spatial heterogeneity in the diet composition, with higher levels of trophic diversity in riverine populations. Water temperature, dissolved oxygen, and conductivity were important environmental factors driving changes in prey taxa of populations from different habitats. The dietary composition of populations in Shuikou Reservoir showed significant seasonal heterogeneity, with summer being the season with the highest level of trophic diversity. Total nitrogen, turbidity degree, pH, and permanganate index were the important environmental factors driving the prey taxa changes of populations in different seasons of Shuikou Reservoir. Our results indicated that *C. zillii* are omnivorous; they have a wide range of recipes in both rivers and reservoirs in southern China, and show high trophic plasticity in different habitats and at different seasons of the year.

## Graphical abstract

The feeding patterns of invasive populations of *C. zillii* in different habitats and seasons within a large‐scale geographic area (spanning 9.86° latitude and 10.47° longitude) in China were directly compared.

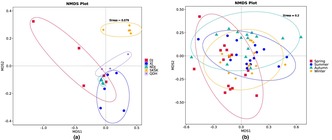

## 1 Introduction

Biological invasions are recognized as a major driver of biodiversity loss on a global scale (Butchart et al. 2010), placing severe stresses on terrestrial, freshwater, and marine ecosystems, with widespread ecological and economic impacts (Simberloff et al. 2013). Freshwater ecosystems have the highest concentration of species per unit surface area on Earth (Dudgeon et al. 2006); however, it is also one of the ecosystems most heavily invaded by exotic species (Moyle 2001). Currently, 277 invasive alien freshwater species have been reported globally, of which fishes are the largest taxonomic group (161 species in total) (Corrales et al. 2020). Among the various pernicious invasive fish species globally, tilapias are some of the most invasive and threatening taxa (Ortega et al. 2015). Tilapias (Osteichthyes, Cichlidae) belong to the family Cichlidae, which is a collective name for many species in the genera *Coptodon*, *Oreochromis*, *Pelmatolapia*, and *Sarotherodon*, and mainly includes redbelly tilapia (*Coptodon zillii*), Mozambique tilapia ( *Oreochromis mossambicus* ), Nile tilapia ( *O. niloticus* ), blue tilapia ( *O. aureus* ), and Mango tilapia ( *Sarotherodon galilaeus* ). Currently, *C. zillii* is one of the most widely introduced and dangerous tilapias in the world (Canonico et al. 2005).

*Coptodon zillii*, formerly known as *Tilapia zillii* , is native to the northern half of Africa and some parts of the Middle East (Philippart and Ruwet 1982). Since the beginning of the 20th century, redbelly tilapia has been introduced intentionally and unintentionally to various parts of the world as a biocontrol agent against aquatic weeds or as an aquaculture object, and by virtue of its strong environmental adaptability and distinctive life‐history traits, it has become a naturalized species in many countries around the world (Geletu et al. 2024).

*Coptodon zillii* is one of the cold‐tolerant tilapia species, which is considered a potential competitor of native fishes in food and spawning areas (Costa‐Pierce 2003). In recent years, several countries including the United States (Cassemiro et al. 2018), Iran (Bavali et al. 2022), Iraq (Mohamed and Al‐Wan 2020), Japan (Ishikawa and Tachihara 2008) and China (Gu et al. 2016) have reported negative impacts (e.g., degradation of water quality and reduction of native fish communities) of *C. zillii* on native fish species and ecosystems. *C. zillii* has been included as one of the invasive alien species in the Global Invasive Species Database (https://www.iucngisd.org/gisd/) (2024). China is one of the countries most seriously jeopardized by the invasion of *C. zillii*, which was introduced into the country from Thailand by the Guangdong Food Branch of the China National Cereals and Oils Import and Export Corporation in 1978 (Cai 1979) and was cultured in the coastal provinces of China, including Guangdong, Fujian, and Guangxi, in the 1980s and 1990s. Because of its small size and slow growth rate, this fish is usually not popular among farmers, and the main purpose of introducing this species was to cross it with Mozambique tilapia to produce hybrids with strong cold hardiness (Liao and Liu 1989). As a result, it was soon abandoned by farmers and gradually escaped into natural water bodies due to prolonged unmanaged conditions. Due to its low‐temperature tolerance, adaptability to the environment, and high fecundity, this species has gradually established populations in the Pearl River system (e.g., Hongshui River, Beijiang River, Dongjiang River and Xijiang River, etc.), the southeastern coastal system (e.g., Hanjiang River, Minjiang River and Qiantang River, etc.), the rivers of Hainan Island (e.g., Nandujiang River, Wanquan River, and Changhua River) and Yangtze River systems (e.g., Jinsha River and Xiangjiang River) and has shown a tendency for further expansion. *C. zillii* poses a serious threat to the structure and function of Chinese aquatic ecosystems, and on December 20, 2022, *C. zillii* was included in the List of Invasive Species for Priority Management by the Chinese government (Gu, Jia, et al. 2023; Gu, Luo, et al. 2023), making it one of the 59 most invasive exotic species in China. Under the background of economic globalization, climate change, and increasing human activities, if no active and reasonable management measures are taken, this species is bound to spread further in China's aquatic ecosystems and cause outbreaks, posing further threats to aquatic biodiversity. Analyzing the biological mechanism of the successful invasion of *C. zillii* is a prerequisite for its prevention and scientific control.

Previous studies have shown that key factors in the success of fish invasions include propagule pressure and genetic variation (Drolet and Locke 2016), their pre‐adaptation to invasive environmental conditions (Wang et al. 2020), and their unique life‐history traits such as tolerance to environmental change (Christensen et al. 2021), rapid growth (Budy et al. 2013), reproduction (Mouchlianitis et al. 2022) and trophic plasticity (Cathcart et al. 2019). Trophic plasticity, i.e., the ability to change diets, which is often discussed in terms of diet breadth, is a necessary assumption for understanding the success of invasions of exotic species (Simon and Townsend 2003), and is generally supported by empirical evidence. For example, extensive trophic plasticity has facilitated the successful invasion of bleak ( *Alburnus alburnus* ) into freshwater waters throughout the Iberian Peninsula (Almeida et al. 2017). As another example, the Eastern mosquitofish ( *Gambusia holbrooki* ), native to the southeastern United States, is an adaptable omnivore and a generalist predator, able to change its diet according to food availability in different habitats (both lotic and lentic) in northwestern Turkey (Saç 2023).

Previous analyses of *C. zillii* in its native and invasive ranges have shown that it has a broad diet, which varies with food availability, season, and environmental conditions (Philippart and Ruwet 1982; Spataru 1978; Mohamed and Al‐Wan 2020). Under natural conditions in its native range, *C. zillii* feeds mainly on leaves, stems, and root systems of large aquatic plants, as well as on terrestrial plant leaves that fall into the water, filamentous algae, and diatoms, with diatoms comprising more than 80% of its food (Philippart and Ruwet 1982). In the absence of aquatic plants, *C. zillii* also feeds on blue‐green algae, zooplankton, crustaceans, insects, and fish eggs (Philippart and Ruwet 1982). In Lake Kinneret, Israel, *C. zillii* feeds mainly on algae, insects, insect larvae, and pupae in winter and spring, and zooplankton in summer and fall (Spataru 1978; Philippart and Ruwet 1982). After invasion into the Garmat Ali River, Iraq, detritus (44.6%) made up the highest percentage of its diet, followed by algae (19.9%), macrophytes (19.7%), and diatoms (13.3%) (Mohamed and Al‐Wan 2020). In view of this, trophic plasticity may be one of the major factors in the successful invasion of *C. zillii*.

So far, although a small number of studies have been carried out on the feeding ecology of *C. zillii* in either its native or invasive range, these studies have focused only on very specific sites at small geographic scales, such as the Shadegan Wetland in Iran (Bavali et al. 2022), the Garmat Ali River in Iraq (Mohamed and Al‐Wan 2020) and the AlTharthar Arm–Tigris River in Iraq (Wahab 2021) within its invasive range, and Lake Kinneret in Israel (Spataru 1978), Lake Nasser in Egypt (Shalloof et al. 2020) and the Otamiri River in Nigeria (Agbabiaka 2012) in its native range. In particular, within its invasive ranges, there remains a lack of studies on dietary plasticity at large geographic scales. Moreover, in terms of geomorphology, hydrodynamics, and biological characteristics, river mainstem (lotic) and reservoir (lentic) habitats can play an active role in fish feeding strategies by providing different food supplies (Garcia et al. 2018). To date, there is still a lack of data on how habitat type (rivers, reservoirs), seasons, and environmental characteristics influence the feeding ecology of *C. zillii*; however, understanding the feeding habits of this species and the role of associated habitat characteristics is critical to interpreting food web dynamics and resource allocation, as well as determining appropriate management and control strategies.

Typically, morphological identification of stomach contents is the most common method used in the analysis of fish feeding habits (Dahl et al. 2017). However, there are limitations to this approach; the plasticity and genetic variability of morphological phenotypes can lead to identification errors; in addition, many populations of organisms have cryptic taxa that cannot be accurately identified by morphological methods, and sex and developmental stage can similarly affect morphological identification, and the digestive process renders many samples visually unrecognizable, which all lead to identification errors (Pompanon et al. 2012). Recently, the method of DNA metabarcoding (Pompanon et al. 2012) has provided powerful technical support for dietary studies, which can solve the difficulties that cannot be overcome by traditional morphological identification and is faster and more accurate, and has been applied to the study of fish feeding habits (Harms‐Tuohy et al. 2016).

In order to gain a more comprehensive understanding of the trophic plasticity of *C. zillii* within the invasion range in China, we collected samples of stomach contents from five representative water bodies within a large‐scale geographic area spanning 9.86° latitude and 10.47° longitude, and at the same time, the sampled water bodies belong to two different habitat types (rivers and reservoirs), which represented both lotic and lentic habitats. Furthermore, for a specific habitat (reservoir), we chose four seasons (spring, summer, fall, and winter) as the study period because the food supply in the reservoir during the four seasons could completely cover the food spectrum of *C. zillii*. We used DNA metabarcoding technology to characterize the diet items of stomach contents, and thus better understand the extent of variation in the diet of *C. zillii*. Our objectives were to assess: (1) the overall plasticity of the feeding habits of *C. zillii*; (2) whether there is size (standard body length) specificity and population specificity in feeding habits, i.e., is there a significant difference in feeding habits among samples of different body lengths and populations? (3) Do differences in habitat environmental, seasonal, and climatic parameters explain differences in feeding habits (prey diversity, frequency of occurrence (FO), relative abundance of diet items, etc.) among populations? Based on previous studies, we predicted that (1) *C. zillii* will be omnivores with a high dependence on phytoplankton and zooplankton, (2) there will be significant differences in feeding habits among body sizes (standard lengths) and among populations, and (3) some of the inter‐population differences in feeding habits may be related to differences in local environmental and climatic factors. To the best of our knowledge, this is the first study that directly compares the feeding patterns of invasive populations of *C. zillii* within a large‐scale geographic area and among different habitats.

## 2 Materials and Methods

### 2.1 Study Area

**FIGURE 1:**
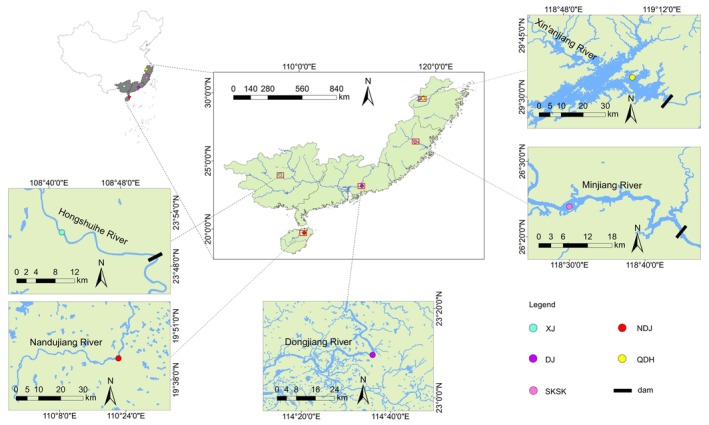
Map of the sampling sites of *Coptodon zillii* in China. The locations of QDH, SKSK, DJ, NDJ, and XJ were indicated with yellow, pink, purple, red, and light blue dots, respectively.

The five sampling sites selected in this study were the water bodies that were seriously invaded by *C. zillii* in China, and they represented most of the invaded areas of *C. zillii* in China, which were, from north to south, Qiandao Lake (QDH), Shuikou Reservoir (SKSK), Letan Reservoir (XJ), Dongjiang River (DJ), and Nandujiang River (NDJ), of which QDH, SKSK, and XJ represented the reservoir (lentic) habitat, while DJ and NDJ represented river habitat, and information about the sampling sites is shown in Figure 1.

Qiandao Lake, located in Chun'an county, Zhejiang Province, is a valley‐type reservoir in the upper reaches of the Qiantang River. The reservoir has an average depth of 30 m and a water area of 580 km^2^. It's a subtropical reservoir, which is the northernmost part of the invasive range of *C. zillii* in China. The origin of *C. zillii* in Qiandao Lake is unknown, but as early as 1987, Nile tilapia was cultured in hapa nets in this reservoir by the Aquatic Bureau of Chun'an county (Zhou 1988). We hypothesized that the *C. zillii* were probably unintentionally introduced into hapas by mixing with Nile tilapia fry and then escaped from the hapas into the reservoir.

Shuikou Reservoir, located in Yanping District, Nanping City, Fujian Province, is a river‐type reservoir in the middle reaches of the Minjiang River. It covers an area of about 99.6 km^2^ with an average water depth of about 65 m. Since the impoundment of the reservoir in March 1993, *C. zillii* have been farmed in earthen ponds around the reservoir on a large scale. They escaped into the reservoir due to the poor management of farmers. The number of *C. zillii* in the reservoir increased year by year, and a large population was established since 2010 (He et al. 2013).

Located in Xincheng County, Laibin City, Guangxi Zhuang Autonomous Region, Letan Reservoir is a large hydropower station on the lower reaches of the Hongshuihe River in the Pearl River Basin. The reservoir has an average water depth of 112 m and a total capacity of 9.5 × 10^8^ m^3^. In 2007, *C. zillii* was introduced and cultured in hapas in the upper reaches of the Hongshuihe River. Gradually, they escaped from the hapas and established populations throughout the upper reaches of the Hongshuihe River. The *C. zillii* in the Letan Reservoir may have spread from the upper reaches.

The DJ sampling site was located in Huizhou City, Guangdong Province, in the middle reaches of the Dongjiang River, which is one of the main streams of the Pearl River system. The Dongjiang River has a total length of 562 km and a watershed area of 35,340 km^2^. *C. zillii* was first cultured in Guangdong province after its introduction from Thailand in 1978, and due to the lack of management, it soon escaped into the natural water bodies. Owing to the well‐developed network of the Pearl River system in Guangdong province, *C. zillii* established its population in the Dongjiang River very quickly. In recent years, *C. zillii* has expanded rapidly, and it has become a dominant and prevalent species in the Dongjiang River (Gu et al. 2020).

The NDJ sampling site was located in Ding'an County, Hainan Province, in the lower reaches of the Nandujiang River, which is the largest river in Hainan Province, with a main stream length of 334 km and a watershed area of 7033 km^2^. The culture of *C. zillii* in Hainan Province started in 1986, but it was quickly abandoned due to poor farming performance. Consequently, it escaped into natural water bodies and established populations in the Nandujiang River (Gu et al. 2016). In recent years, *C. zillii* has spread rapidly, and it has become a dominant species in the Nandujiang River (Gu et al. 2020).

To obtain the water environment and climate information of the five sampling sites (Table S1), we downloaded nine water environment parameters from the China National Environmental Monitoring Centre (https://www.cnemc.cn/), including water temperature (TEMP), dissolved oxygen (DO), pH, conductivity (COND), turbidity degree (TD), permanganate index (PI), ammonia nitrogen (AN), total phosphorus (TP), and total nitrogen (TN). Additionally, four types of climatic data were downloaded from the Public Meteorological Service Center of the China Meteorological Administration (https://www.weather.com.cn), including daily mean temperature, daily maximum temperature, daily minimum temperature, and daily precipitation sum.

### 2.2 Field Sampling and Laboratory Procedures

#### 2.2.1 Sample Collection and Preparation

Field sampling was conducted in 2 phases, with phase 1 from October to November 2022 at 5 sampling sites, including QDH, SKSK, XJ, DJ, and NDJ. 110 (QDH), 63 (SKSK), 68 (XJ), 51 (DJ), and 55 (NDJ) of live specimens were trapped using gillnets with 6 mesh sizes (mesh sizes of 3–8 cm, respectively). The purpose of the first phase is to assess dietary differences caused by geographical differences and investigate the effects of associated environmental features on feeding habits (Table S1). In phase 2, from March 2023 to February 2024, different sizes of specimens were trapped with gillnets (same mesh size as in phase 1) at SKSK in the middle of each month. The number of live samples collected per month ranged from 80 to 100, for a total of 1079 specimens. In order to accurately assess the effect of season on feeding traits, we divided the sampling period in SKSK into 4 seasons: spring (March to May), summer (June to August), fall (September to November) and winter (December to February). The collection of live specimens at the above two stages was entrusted to experienced local fishermen, and both were licensed by the respective fisheries department of the sampling site. Each trapping was conducted at dawn, which corresponded to the peak feeding time of *C. zillii*.

At each sampling event, once the live fish samples were collected, sex was first determined from the morphology of the genital pore. While it was immediately immersed in an overdose of anesthetic (MS‐222) solution (0.4 mg/mL) for 15 min, followed by measurement of standard length (SL) using digital calipers (to the nearest 0.1 cm) and body weight using an electronic balance (to the nearest 0.1 g). At the end of the measurements, each individual was dissected from the digestive tract, and the whole stomach (esophagus to just below the pyloric valve) was excised. The degree of stomach fullness was determined according to the method of Yin (1995), and the degree of fullness was categorized into six grades (grades 0–5) (Table S2). If the stomach fullness degree was graded from 4 to 5, all stomach contents (200–500 mg) were carefully removed with forceps, preserved in a 1.5 mL centrifuge tube, and immediately put on dry ice to stop its digestion process and preserve the DNA in the stomach contents. If the stomach fullness degree was graded from 0 to 3, no further sampling was done. In stage 1, we collected 23 samples of stomach contents, of which the number of samples from all sampling sites was 5, except for QDH, where the number of samples was 3. In phase 2, we collected 60 samples of stomach contents, with the number of samples collected at each month being five. Information on the standard length, body weight, and sex of the samples from each sampling site is shown in Tables S1 and S3.

#### 2.2.2 DNA Extraction and Sequencing

After transporting the stomach contents samples back to the laboratory on dry ice, 2–20 mL of TE buffer was added to the samples, and the mixture was completely homogenized using a TissueRuptor. Finally, 200 μL of sub‐samples were extracted from the homogenized contents, and the DNA in the samples was extracted using the QIAamp Stool Mini Kit (QIAGEN, Hilden, Germany), with a slight modification of the procedure. The lysis time of the samples in InhibitEx Buffer was extended to 30 min at 72°C. After lysis buffer and proteinase K were added to the samples, the samples were further homogenized with a 10‐min bead‐beating step using 0.70 mm garnet beads (MOBIO, Carlsbad, CA, USA). After the DNA was eluted, the DNA concentration was quantified using an ND‐1000 nanodrop spectrophotometer (Nanodrop Technologies Inc., Wilmington, DE, USA). All DNA samples were diluted with sterile water to a standard concentration of 20 ng/μL. For each extraction, an empty centrifuge tube was taken as a negative control, and three negative controls were randomly selected for sequencing. The diluted DNA was used as a template for PCR amplification against the V4 region of the eukaryotic 18S rRNA gene using primers 528F (5′‐GCGGTAATTCCAGCTCCAA‐3′) and 706R (5′‐AATCCRAGAATTTCACCTCT‐3′) with Barcode (Cheung et al. 2010) (Table S4). The total volume of the PCR reaction system was 20 μL and contained the following: 10 μL Phusion MasterMix (2×), 1 μL forward primer (10 μmol/L), 1 μL reverse primer (10 μmol/L), 1 μL DNA template (20 ng/μL), and 7 μL of sterile double‐distilled water (ddH_2_O). The PCR reaction procedure is as follows: pre‐denaturation at 98°C for 1 min; followed by 30 cycles, each cycle including denaturation at 98°C for 10 s; annealing at 50°C for 30 s; extension at 72°C for 30 s; and extension at 72°C for 5 min at the end of the last cycle. PCR products were detected using agarose gel electrophoresis at a 2% concentration. After 2% agarose gel electrophoresis, the gel containing the target bands was cut and recovered. The PCR products were purified using the Gene JET Gel Recovery Kit (Thermo Scientific). The library was constructed using the Ion Plus Fragment Library Kit 48 rxns (Thermofisher), and after the constructed library was qualified by Qubit quantification and library detection, it was sequenced using Ion S5XL (Thermofisher).

#### 2.2.3 DNA Sequence Processing

Using Cutadapt v1.9.1 software (Jiao et al. 2016), the low‐quality part of the reads was first removed, and then the Barcode and primer sequences were truncated to get the raw data (raw reads). The raw reads were compared with the species annotation database through the Vsearch program (Qin et al. 2012). Chimeric sequences were removed to get the final valid data (clean reads). All clean reads from all samples were clustered using Uparse v7.0.1001 software (Martin 2011), and by default, sequences were clustered into Operational Taxonomic Units (OTUs) with 97% identity, and the sequences with the highest FO in the OTUs were used as representative sequences of the OTUs. After removing the sequences of *C. zillii*, the representative sequences of OTUs were annotated using the RDP Classifier Version 2.2 software (Torbjørn et al. 2016), compared to the Silva132 database (Haas et al. 2011) using the default parameters except that the minimum match value was adjusted to 98%. Also, these assembled sequences were double‐checked in Nucleotide BLAST on Genbank with a sequence identity of greater than or equal to 97%, with an *e*‐value threshold of 10^−5^. The prey composition of each sample was analyzed at each taxonomic level: kingdom, phylum, class, order, family, genus, and species. A rapid multiple sequence comparison was performed using Muscle Version 3.8.31 software (Edgar 2013) to obtain phylogenetic relationships of all OTU sequences. Finally, the data from each sample were homogenized, and all subsequent analyses were based on the homogenized data.

### 2.3 Data Analyses

#### 2.3.1 Analysis of Basic Dietary Data

To determine the adequacy of the amount of sequencing data for each sample and the sample size for each population, the species rarefaction curve and species accumulation boxplot were calculated and plotted using the vegan package and the ggplot2 package in R software (Version 4.0.3). Fractions that could not be identified in each sample (less than 97% sequence homology in Nucleotide BLAST on Genbank) were excluded from analysis.

According to Deagle et al. (2019), the importance of each prey species in the diet was estimated by the FO and relative read abundance (RRA), respectively. FO is the number of samples containing a particular food group as a percentage of the total number of samples and is calculated as follows: $\mathrm{FO}=\frac{N}{T}\times 100$, where *N* is the number of stomachs in which the prey items of one particular category are found and *T* is the total number of stomachs with food in the sample. RRA is the percentage of the number of sequences of a food category to the total number of sequences in that sample, reflecting the relative biomass, and is calculated as follows: $\mathrm{RRA}=\frac{1}{N}\sum_{j=1}^{N}\frac{\mathrm{Si},j}{\sum_{i=1}^{T}\mathrm{Si},j}\times 100$, where *T* is the number of food categories, *N* is the total number of valid samples, *S*_*i,j*_ is the sequence number of food category *i* in sample *j*. Feeding strategies (generalized or specialized) were analyzed by plotting the relationship between FO and RRA. When a prey taxon has a low FO but a high RRA, it indicates specialized feeding on a specific prey by *C. zillii*, whereas when the FO is high but the RRA is low, it represents a generalized feeding strategy.

A bar chart of RRA of species was plotted based on the results of species annotation at the phylum level using Perl 5.26.2 software. The top 20 species in terms of RRA of prey were selected, and the rest of the species were set as “other” in the bar chart. Species codes for stomach contents at the phylum level are shown in Table S5.

Shannon‐Wiener index (*H*′), Pielou's evenness index (*J*), and niche breadth index (*B*) (Levins 1968) were used to describe the diet diversity of *C. zillii*. The formula was calculated as follows:

$$H^{\prime }=-\sum_{i=1}^{S}\left(\mathrm{pi}\times \ln\mathrm{pi}\right),J=-\frac{\sum_{i=1}^{S}\left(\mathrm{pi}\times \ln\mathrm{pi}\right)}{\ln S},B=\frac{1}{\sum_{i=1}^{S}{\left(\mathrm{pi}\right)}^{2}}$$

where *H*′ is the Shannon‐Wiener index of prey diversity in the diet and *p* is the proportion of prey items of one particular food category *i* to the total number of prey categories found. *S* denotes the total number of prey items. The *H*′ and *J* were calculated using QIIME (The Quantitative Insights Into Microbial Ecology) software (Version 1.9.1) (Caporaso et al. 2010) to assess dietary diversity within a single sample. Differences in prey diversity between populations were analyzed for significance by the Kruskal‐Wallis rank sum test, and box plots were drawn using the ggplot2 package of R software (Version 4.0.3).

Based on the species annotation results and the abundance information of the feature sequences of all the samples, the information of the feature sequences of the same classification was combined and processed to obtain the species abundance information table (Profiling Table). The phylogenetic relationships between the feature sequences were also utilized to calculate the Weighted unifrac distance and Bray‐Curtis distance between samples using QIIME software (Version 1.9.1) (Caporaso et al. 2010). The Weighted unifrac distance and Bray‐Curtis distance matrix heatmap were plotted using Perl 5.26.2 software. Dietary differences between samples, populations, or seasons were assessed by non‐metric multidimensional scaling (NMDS) based on Weighted unifrac distances in the ade4 and ggplot2 packages of the R software (version 4.0.3). To further analyze the significance of dietary differences between populations or between seasons, a permutational multivariate analysis of variance (PERMANOVA) based on the Bray‐Curtis distance was performed in the vegan package of R software (version 4.0.3). Significant *p*‐values were calculated during 999 random permutations. In order to search for species with statistical differences among recipes of different populations, a histogram of the distribution of LDA values was plotted using the linear discriminant analysis (LDA) effect size method in the online software Galaxy module (http://galaxy.biobakery.org/). Species with LDA scores greater than a set value (the default setting was 4) were statistically different between groups.

To assess dietary overlap among populations, we further calculated the Schoener overlap index (*α*) (Schoener 1970). This overlap measure ranges from 0 (no overlap) to 1 (complete overlap in resource use), and values above 0.6 are considered biologically significant according to Wallace and Ramsey (1983). The formula was calculated as $\alpha =1-0.5\left(\sum_{i=1}^{n}|P_{\mathrm{xi}}-P_{\mathrm{yi}}\right)$; where *α* is the measure of the relative amount of dietary overlap, varying between 0 (no overlap) and 1 (complete overlap), *P*_*xi*_ represents the proportion of food category *i* in the diet of the population *x*, *P*_*yi*_ is the proportion of food category *i* in the diet of the population *y*, and *n* is the number of food categories.

#### 2.3.2 Feeding Patterns as a Function of Size and Population

We use food category‐specific generalized linear models (GLMs) with a binomial error distribution and a logit link function to investigate whether the presence of a prey item in the diet was affected by the size of the fish or the sampling location. In addition, to assess the effects of SL and sampling location on the *H*′, *J*, and *B*, we used GLMs with a gamma error distribution and a log link function.

#### 2.3.3 Effects of Environmental, Geographic, and Climatic Variables on Trophic Patterns of Different Populations

##### 2.3.3.1 Screening for Environmental and Climatic Variables

To remove possible high levels of multicollinearity between the environmental and climatic factors, the linear correlations between the factors were examined using the variance inflation factor (VIF) method. Factors with VIF > 10 were removed, thus excluding highly autocorrelated environmental and climatic factors and retaining those with the greatest influence on diet composition.

##### 2.3.3.2 Spearman's Correlation Analysis

Spearman's correlation coefficients between prey taxa and environmental factors were calculated and tested for significance using the corr.test function of the psych package in R software (version 4.0.3). The results were then visualized using the pheatmap function of the pheatmap package.

##### 2.3.3.3 Analysis of Multiple Linear Regression Models

Multiple linear regression models were used to screen for environmental factors that had significant effects on the *H*′, *J*, and *B*. The explanatory variables were standardized, and the response variables were tested for normal distribution, and those that did not meet the normal distribution were log‐transformed or inverted. Multiple linear regression analysis was completed in SPSS 22.0 software.

##### 2.3.3.4 Redundancy Analysis (RDA)

In order to further illustrate the response of different prey items to various environmental factors, CANOCO 4.5 software was used to analyze the effect of environmental factors on prey items of populations in various habitats or different seasons by means of gradient analysis. Firstly, the RRA data of prey species of each sample were transformed by Hellinger transformation, and then detrended correspondence analysis (DCA) was performed. The sorting method was decided according to the lengths of gradient (LGA) in the first axis of the analyzed results. RDA was used when the LGA was less than 3, and canonical correspondence analysis (CCA) was used when the LGA was greater than 4. Both are used when the LGA was between 3 and 4 (Ter Braak and Smilauer 2002). RDA was chosen because the LGA in the first axis in the DCA analysis was 3.57 (between different habitats) (Table S6) and 3.21 (between different seasons in SKSK) (Table S6), respectively.

During the RDA analysis, RRA data for prey species were used as the response variable, and environmental factors were used as predictor variables. Predictor variables were not transformed because the significance of the RDA results did not depend on the parameter distribution assumptions of the predictor variables (Ter Braak 1986). Rare species (i.e., species ranked lower than the 20th percentile in RRA in the diet for each population) were included in the analysis because they contribute to the breadth of taxa consumed by *C. zillii*, inform our findings of species sensitive to invasion, and removal may have affected the results (Poos and Jackson 2012). Monte Carlo permutation tests (*n* = 5000) were used to assess significance between the RDA ordination axes for prey species and environmental variables, and the first two axes were used to plot the two‐dimensional ordination of sample–environmental factors.

##### 2.3.3.5 Mantel Test

Finally, we investigated whether the Bray‐Curtis distance for dietary differences and dietary overlap between pairs of populations was related to geographic distance between them. Using the Mantel test, we first developed pairwise geographic distance matrices for all sampling points based on GPS coordinates and compared them against pairwise matrices of Bray‐Curtis distances and Shoener's overlap index values, respectively, by completing 10,000 permutations in the ade4 R package (v1.7‐15).

## 3 Results

### 3.1 Feeding Intensity

Stomach fullness of five population samples from two habitats showed that the DJ population had the highest Fullness Index (64.71%) and the SKSK population had the highest Empty Index (60.32%) (Table S7). There was a significant seasonal difference in stomach fullness of the SKSK population (Table S7), and the seasonal pattern of the fullness index was: spring (87.68%) > summer (56.84%) > fall (32.22%) > winter (31.40%), indicating that the feeding intensity was significantly higher in spring and summer than in fall and winter.

### 3.2 DNA Sequencing

The average number of effective tags of stomach contents samples from five populations from two habitats ranged from 92,286 to 130,330, and the percentage of effective tags reached 86.89%–97.65% (Table S8). The average number of effective tags of stomach contents samples from four seasons of SKSK ranged from 79,908 to 99,437, and the percentage of effective tags reached 91.17%–94.49% (Table S8). The quality of sequencing data of all samples met the requirements for analysis.

As the sequencing depth increased, the rarefaction curve of the Shannon‐Wiener index tended to flatten out for each sample (Figure S1), indicating that the sequencing data volume was leveling off and that more data volume did not have a significant effect on the Shannon‐Wiener index. As the sample size increased, the position of the boxplots increased sharply, indicating that a large number of species were found in the samples, but the boxplots did not reach a plateau for either the five populations from different habitats or the samples from different seasons in SKSK (Figure S2), suggesting that the sample size used for the study was insufficient.

### 3.3 Prey Item Composition

475 OTUs were delineated in samples of the five populations from 2 habitats (Table S8), with a total of 22 phyla annotated (Table 1). 2590 OTUs were delineated in samples of the four seasons from the SKSK (Table S8), with a total of 37 phyla annotated (Table 2). In addition, there were some OTUs that failed to be attributed to known taxonomic units. All prey annotated to the phylum level were grouped into 5 food categories: phytoplankton, protozoa, zooplankton, zoobenthos, and detritus. The mean RRA of phytoplankton, protozoa, zooplankton, zoobenthos, and detritus in the diet of the five populations from two habitats was 52.84%, 8.79%, 12.95%, 3.12%, and 13.49%, respectively (Table 1). The mean RRA of phytoplankton, protozoa, zooplankton, zoobenthos, and detritus in the diet of seasonal samples from SKSK was 10.68%, 31.27%, 10.35%, 21.42%, and 15.62%, respectively (Table 2). It can be inferred that *C. zillii* is omnivorous and the proportions of various food categories in its diet varied with habitat and season. The relationship between FO and RRA showed (Tables 1 and 2, Figures S3 and S4) that generalist trophic strategies were dominant in the *C. zillii* populations.

**TABLE 1:** Diet composition of *Coptodon zillii* between habitat types by frequency of occurrence (FO, %) and relative read abundance (RRA, %).

Food category	Classification	Habitat type									
		River				Reservoir					
		DJ		NDJ		QDH		SKSK		XJ	
		FO	RRA	FO	RRA	FO	RRA	FO	RRA	FO	RRA
Phytoplankton	Chlorophyta	60	25.89	100	85.47	100	32.98	100	2.74	100	24.42
	Bacillariophyta	60	3.37	20	0.42	100	24.91	100	63.16	40	0.84
Protozoa	Ciliophora	20	0.21	80	3.58	0	0	100	14.32	100	17.05
	Microsporidia	20	4.42	0	0	0	0	0	0	0	0
	Cercozoa	0	0	80	1.89	0	0	20	0.21	0	0
	Picozoa	0	0	0	0	33.33	0.35	20	0.42	0	0
	Apicomplexa	40	0.63	20	0.21	0	0	20	0.21	20	0.42
Zooplankton	Arthropoda	80	43.58	80	4.63	33.33	0.35	60	1.05	80	9.47
	Rotifera	20	0.21	40	0.42	0	0	80	1.26	60	2.32
	Cnidaria	0	0	0	0	0	0	20	1.26	20	0.21
Zoobenthos	Bryozoa	0	0	0	0	33.33	0.70	20	10.11	0	0
	Annelida	0	0	0	0	66.67	2.46	20	0.21	0	0
	Nemertea	20	0.21	0	0	0	0	0	0	20	0.63
	Gastrotricha	0	0	0	0	0	0	0	0	20	0.42
	Nematoda	0	0	0	0	0	0	20	0.42	20	0.21
	Mollusca	0	0	0	0	0	0	20	0.21	0	0
Detritus	Ascomycota	60	5.68	40	0.63	100	21.05	40	0.63	100	16.63
	Streptophyta	60	2.32	20	0.42	33.33	4.91	0	0	60	4.00
	Oomycota	0	0	20	0.21	0	0	20	0.21	40	1.89
	Basidiomycota	20	0.42	0	0	33.33	0.70	0	0	80	1.68
	Cryptomycota	40	1.05	40	0.63	33.33	1.05	20	0.21	40	0.84
	Chytridiomycota	0	0	0	0	33.33	0.35	80	0.84	40	1.05
Others	Unidentified	80	12.00	100	1.47	100	10.18	100	2.53	100	17.89

**TABLE 2:** Diet composition of *Coptodon zillii* between seasons in SKSK Reservoir by frequency of occurrence (FO, %) and relative read abundance (RRA, %).

Food category	Classification	Season							
		Spring		Summer		Autumn		Winter	
		FO	RRA	FO	RRA	FO	RRA	FO	RRA
Phytoplankton	Chlorophyta	80	2.013	100	3.30	86.67	2.96	93.33	1.35
	Bacillariophyta	86.67	22.89	73.33	0.48	73.33	0.26	93.33	9.42
	Haptophyta	33.33	0.021	13.33	0.015	0	0	0	0
Protozoa	Ciliophora	93.33	15.71	100	27.27	100	45.36	100	28.0026
	Microsporidia	0	0	13.33	0.013	0	0	0	0
	Cercozoa	73.33	0.97	73.33	0.45	80	1.92	86.67	0.88
	Apicomplexa	53.33	0.13	73.33	0.77	66.67	0.43	46.67	0.046
	Picozoa	26.67	0.028	0	0	0	0	0	0
	Euglenozoa	26.67	0.21	40	1.39	13.33	0.023	26.67	0.041
	Imbricatea	26.67	0.19	0	0	0	0	6.67	0.010
	Heterolobosea	20	0.031	33.33	0.36	6.67	0.0051	0	0
	Endomyxa	33.33	0.20	0	0	0	0	0	0
	Tubulinea	40	0.21	33.33	0.064	13.33	0.0077	6.67	0.013
	Perkinsozoa	46.67	0.067	20	0.021	53.33	0.20	13.33	0.013
	Foraminifera	20	0.026	0	0	0	0	0	0
	Evosea	13.33	0.021	20	0.010	0	0	0	0
	Discosea	13.33	0.018	0	0	0	0	6.67	0.0077
Zooplankton	Arthropoda	80	5.73	66.67	1.29	86.67	8.018	80	1.82
	Rotifera	73.33	1.36	66.67	4.74	100	8.96	93.33	5.99
	Cnidaria	86.67	1.37	40	0.26	26.67	0.090	60	1.76
Zoobenthos	Bryozoa	40	3.52	33.33	0.41	13.33	0.018	46.67	0.77
	Annelida	80	7.36	100	26.041	93.33	8.018	86.67	18.054
	Nematoda	33.33	0.25	53.33	0.46	60	1.35	53.33	0.085
	Gastrotricha	40	0.082	33.33	0.031	6.67	0.0026	33.33	0.049
	Mollusca	66.67	0.60	100	5.80	93.33	3.073	33.33	4.59
	Platyhelminthes	60	0.34	80	1.90	60	1.28	86.67	1.11
	Tardigrada	26.67	0.031	20	0.17	6.67	0.0051	13.33	0.010
	Porifera	6.67	0.0026	6.67	0.0077	33.33	0.077	33.33	0.18
Detritus	Streptophyta	80	4.76	86.67	8.062	80	2.046	53.33	2.76
	Ascomycota	93.33	12.73	86.67	3.32	66.67	0.33	60	15.46
	Oomycota	66.67	1.055	66.67	0.34	46.67	0.41	53.33	0.21
	Basidiomycota	60	0.74	73.33	2.098	60	0.30	33.33	0.059
	Cryptomycota	73.33	0.36	93.33	1.32	80	3.53	80	0.52
	Chytridiomycota	80	0.79	80	0.21	60	0.17	80	0.14
	Mucoromycota	46.67	0.27	33.33	0.13	46.67	0.077	40	0.12
	Blastocladiomycota	20	0.021	13.33	0.010	6.67	0.12	0	0
	Zoopagomycota	0	0	0	0	6.67	0.0051	0	0
Others	Unidentified	100	15.93	100	9.26	100	10.94	100	6.53

### 3.4 Feeding Habits Variation With Habitat

12, 11, 16, 11, and 17 phyla of food categories were identified in the stomach contents of DJ, NDJ, XJ, QDH, and SKSK populations, respectively (Table 1, Figure S3). Chlorophyta was the food category with the highest RRA in the NDJ, XJ, and QDH populations. Arthropoda and Bacillariophyta were the food categories with the highest RRA in the DJ and SKSK populations, respectively (Figure S3).

The prey diversity indices (*H*′, *J*) in both XJ and NDJ populations were significantly (*p* < 0.05) higher than those in QDH and SKSK populations (Table 3, Figure S5). The Niche breadth index (B) in the XJ population was significantly (*p* < 0.05) higher than those in the other four populations (Table 3).

**TABLE 3:** Trophic diversity (Shannon‐Wiener index [*H*′], Pielou's evenness index [*J*], Niche breadth index [*B*]) of *Coptodon zillii* between habitat types and seasons.

Index	Habitat type					Seasons of SKSK			
	River		Reservoir			Spring	Summer	Autumn	Winter
	DJ	NDJ	QDH	SKSK	XJ				
*H*′	3.11 ± 0.83^bc^	4.85 ± 0.060^a^	2.68 ± 0.71^b^	2.50 ± 0.24^b^	4.53 ± 0.21^ac^	3.81 ± 0.33	4.22 ± 0.30	4.11 ± 0.21	4.18 ± 0.27
*J*	0.71 ± 0.10^bc^	0.90 ± 0.0046^ac^	0.57 ± 0.11^b^	0.55 ± 0.042^b^	0.90 ± 0.020^a^	0.54 ± 0.037	0.61 ± 0.032	0.61 ± 0.021	0.60 ± 0.031
*B*	2.28 ± 0.56^a^	1.37 ± 0.091^a^	1.94 ± 0.40^a^	2.026 ± 0.34^a^	4.25 ± 0.49^b^	2.99 ± 0.47	3.45 ± 0.47	3.065 ± 0.41	2.65 ± 0.31

*Note:* Results are represented by mean ± SE. Significant differences (Kruskal–Wallis rank sum tests) between habitat types are indicated by superscript with different lowercase letters.

**FIGURE 2:**
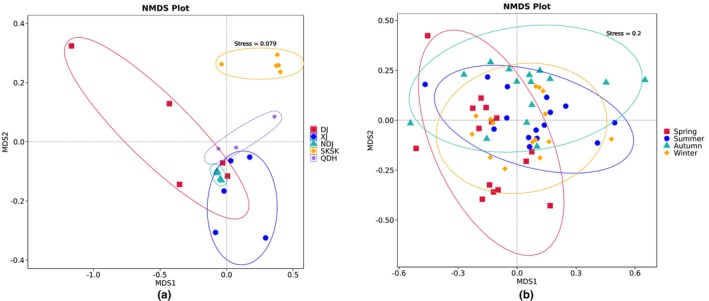
Non‐metric multidimensional scaling ordination (NMDS) of prey items for *Coptodon zillii* among five sampling locations (a) and among four seasons of SKSK (b) based on Weighted Unifrac distance. Each point in the graph represents a sample, and the distance between points represents the degree of difference. Samples in the same group are represented by the same color. When the Stress is less than 0.2, it indicates that NMDS can accurately reflect the degree of difference between samples.

**FIGURE 3:**
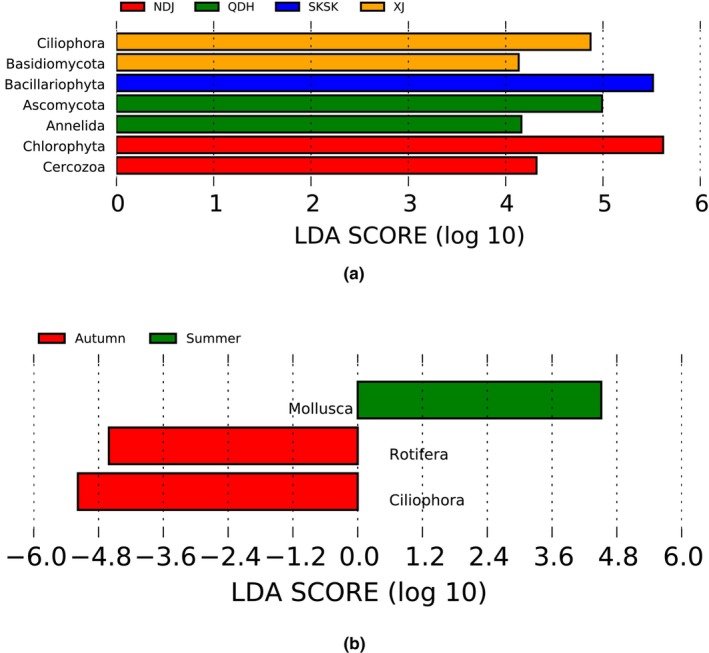
LDA score distribution bar chart based on prey item differences for *Coptodon zillii* among five sampling locations (a) and among four seasons of SKSK (b). The LDA score distribution bar chart shows the species with LDA scores greater than the set value (default set to 4), which are Biomarkers showing statistical differences between groups. The length of the bar chart denotes the impact of different species (i.e., LDA score). The phylum names designated by English letters in the figure are shown in the legend on the right or left.

Extremely significant (*p* < 0.001) Bray‐Curtis distances were detected among six pairwise populations (Table S9). NMDS ordination showed that the coefficient of stress was 0.079 (Figure 2a). PERMANOVA analysis showed that there were significant differences (*p* < 0.05) in diet composition among all paired populations (Table 4). In addition, Schoener's dietary overlap indices between populations ranged from 0.11 to 0.58 (Table S10), which were all less than the threshold (0.6). Histograms of the distribution of the LDA values showed that prey taxa causing significant differences in the diets of the five populations from the different habitats were Chlorophyta, Bacillariophyta, Cercozoa, Ciliophora, Annelida, Basidiomycota, and Ascomycota (Figure 3a).

**TABLE 4:** Results of permutational multivariate analysis of variance (PERMANOVA) applied to diet data for *Coptodon zillii* between habitat types and seasons.

Type of grouping	Pairwise comparison group	df	SumsOfSqs	MeanSqs	*F*.Model	*R* ^2^	Pr (> *F*)
Habitat	DJ–XJ	1 (8)	0.72 (3.083)	0.72 (0.39)	1.87	0.19 (0.81)	0.034
	DJ–NDJ	1 (8)	1.25 (1.78)	1.25 (0.22)	5.63	0.41 (0.59)	0.007
	DJ–SKSK	1 (8)	1.43 (2.13)	1.43 (0.27)	5.36	0.40 (0.60)	0.007
	DJ–QDH	1 (6)	0.71 (2.39)	0.71 (0.40)	1.79	0.23 (0.77)	0.023
	XJ–NDJ	1 (8)	0.86 (1.92)	0.86 (0.24)	3.58	0.31 (0.69)	0.008
	XJ–SKSK	1 (8)	1.36 (2.27)	1.36 (0.28)	4.79	0.37 (0.63)	0.012
	XJ–QDH	1 (6)	0.65 (2.54)	0.65 (0.42)	1.54	0.20 (0.80)	0.025
	NDJ–SKSK	1 (8)	2.012 (0.97)	2.012 (0.12)	16.62	0.68 (0.32)	0.01
	NDJ–QDH	1 (6)	1.17 (1.24)	1.17 (0.21)	5.67	0.49 (0.51)	0.01
	SKSK–QDH	1 (6)	0.98 (1.58)	0.98 (0.26)	3.72	0.38 (0.62)	0.018
Season	Spring–Summer	1 (28)	1.22 (11.91)	1.22 (0.43)	2.87	0.093 (0.91)	0.001
	Spring–Autumn	1 (28)	1.72 (11.24)	1.72 (0.40)	4.28	0.13 (0.87)	0.001
	Spring–Winter	1 (28)	1.15 (12.089)	1.15 (0.43)	2.67	0.087 (0.91)	0.001
	Summer–Autumn	1 (28)	1.19 (10.98)	1.19 (0.39)	3.040	0.098 (0.90)	0.001
	Summer–Winter	1 (28)	0.83 (11.83)	0.83 (0.42)	1.97	0.066 (0.93)	0.003
	Autumn–Winter	1 (28)	0.88 (11.16)	0.88 (0.40)	2.22	0.074 (0.93)	0.013

*Note:* The values corresponding to the residual term are enclosed in parentheses.

Abbreviations: df, degrees of freedom; *F*.Model, *F*‐test value; MeanSqs, mean squares; Pr, *p*‐value; *R* ^2^, the explanatory power of different groups on sample differences, that is, the ratio of group variance to total variance; SumsOfSqs, sum of squared deviations.

### 3.5 Temporal Variation of Feeding Habits

35 (spring), 31 (summer), 29 (fall), and 28 (winter) phyla of food categories were identified in the seasonal samples of the SKSK population (Table 2). Ciliophora was the food category with the highest RRA in summer, fall, and winter (Figure S4). Bacillariophyta was the food category with the highest RRA in spring (Figure S4). No significant (*p* > 0.05) differences in prey diversity indices (*H*′, *J*, *B*) were detected between seasons (Table 3, Figure S5).

Significant Bray‐Curtis distances were detected among four pairwise seasons (*p* < 0.05) (Table S11). NMDS ordination showed that the coefficient of stress was 0.2 (Figure 2b). The PERMANOVA analysis showed that there were significant differences (*p* < 0.05) in diet composition between all paired seasons (Table 4). In addition, the Schoener diet overlap indices between seasons ranged from 0.50 to 0.74 (Table S12), and four pairwise diet overlap indices between seasons exceeded the threshold (0.6). The histogram of the distribution of the LDA values showed that prey taxa with significant differences in the diets of samples among different seasons were Rotifera, Ciliophora, and Mollusca (Figure 3b).

### 3.6 Feeding Patterns in Relation to Standard Length

Generalized linear model analyses revealed no significant effects (*p* > 0.05) of SL, population, and its interactions on the presence and absence of the top 10 prey species in terms of RRA, *H*′, *J*, and *B*.

### 3.7 The Driving Factors of Dietary Differences Between Different Habitats and Seasons

A total of three environmental factors with VIF values less than 10 were detected in samples from two different habitats, i.e., TEMP (VIF = 1.52), DO (VIF = 1.11), and COND (VIF = 1.59). A total of six environmental factors with VIF values less than 10 were detected in seasonal samples from SKSK, i.e., pH (VIF = 3.47), COND (VIF = 4.00), TD (VIF = 4.00), PI (VIF = 7.43), TP (VIF = 3.66), and TN (VIF = 6.23).

Spearman's correlation analysis showed that among the diet of five populations from two habitats (Figure S6), Cercozoa had a highly significant (*p* < 0.01) positive correlation with TEMP and a very significant (*p* < 0.01) negative correlation with COND. Chlorophyta showed a significant (*p* < 0.05) positive correlation with DO. Ciliophora had a very significant (*p* < 0.01) negative correlation with DO. Rotifera showed a significant (*p* < 0.05) negative correlation with DO. Both Ascomycota and Basidiomycota were very significantly (*p* < 0.01) positively correlated with COND. In the diet of seasonal samples from SKSK (Figure S6), Bacillariophyta showed a highly significant (*p* < 0.01) positive correlation with pH and TN. Rotifera was very significantly (*p* < 0.01) negatively correlated with TD and TN, and very significantly (*p* < 0.01) positively correlated with COND. Both Cercozoa and Euglenozoa had a very significant (*p* < 0.01) negative correlation with pH. Euglenozoa showed a significant (*p* < 0.05) negative correlation with TD. Bacillariophyta was significantly (*p* < 0.05) negatively correlated with TD. Ciliophora showed a significant (*p* < 0.05) negative correlation with TN.

As for five populations from two different habitats, the multiple linear regression coefficients between prey diversity indices (*H*′ and *J*) and TEMP as well as COND were very significant (*p* < 0.01) (Table S13). The regression coefficient between *B* and COND was highly significant (*p* < 0.01) (Table S13). As for seasonal samples from SKSK, the regression coefficient between *J* and TP was significant (*p* < 0.05) (Table S14).

**FIGURE 4:**
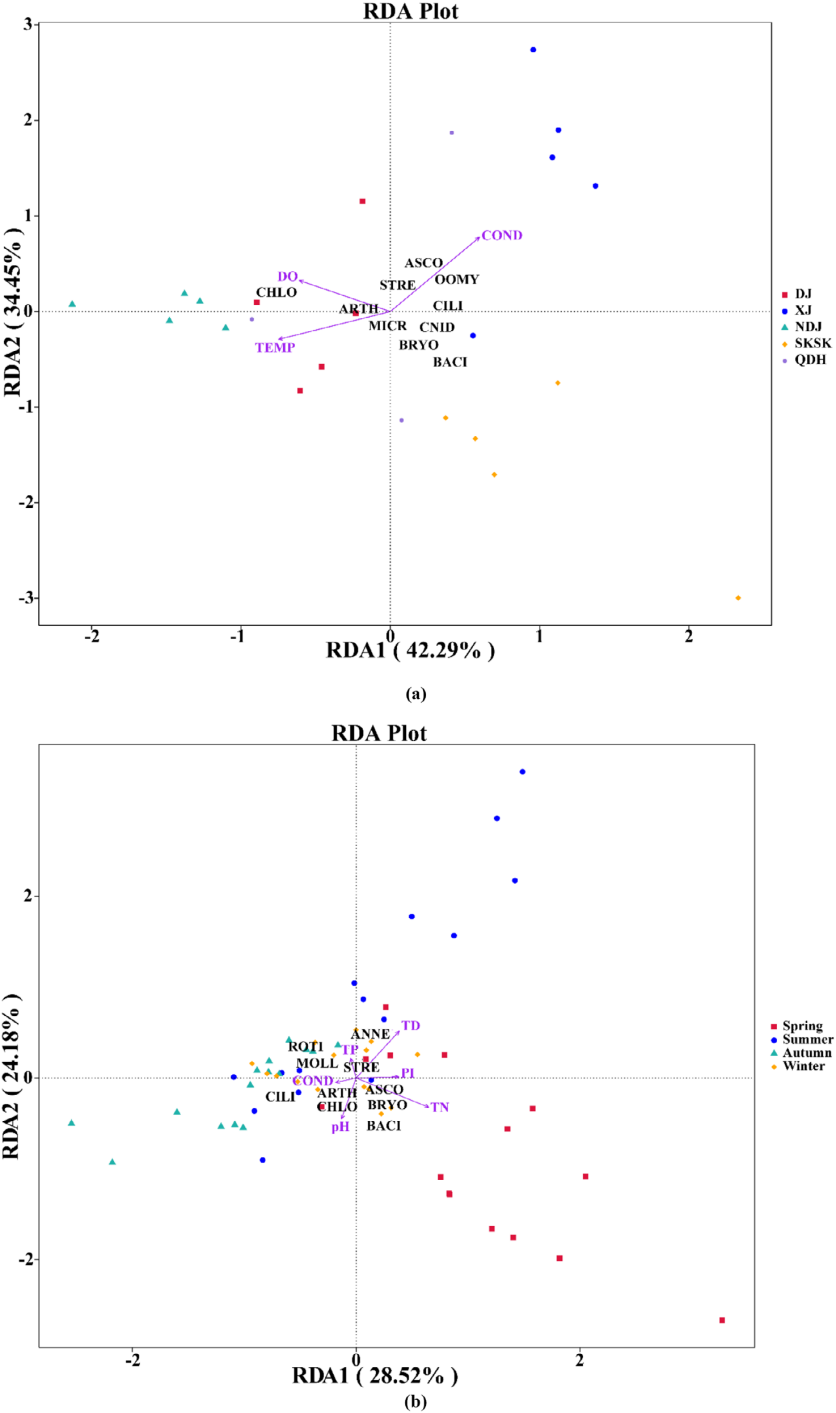
Triplot of redundancy analysis (RDA) for *Coptodon zillii*. (a) RDA integrating top 10 prey taxa, environmental variables, and sites among five sampling locations. Samples from different populations are denoted by different colors (see legend). The main axis (RDA1, horizontal) explains 42.29% of the variance (*F* = 10.2; *p* = 0.002), while the secondary axis (RDA2, vertical) explains 34.45% of the variance (*F* = 4.7; *p* = 0.002). (b) RDA integrating top 10 prey taxa, environmental variables, and seasons in SKSK. Samples from different seasons are denoted by different colors (see legend). The main axis (RDA1, horizontal) explains 28.52% of the variance (*F* = 7.3; *p* = 0.004), while the secondary axis (RDA2, vertical) explains 24.18% of the variance (*F* = 3.3; *p* = 0.002). The environmental variables are shown as arrows. The vector orientations indicate the direction of strongest change, and vector lengths correspond to relative importance. Arrows indicate the relationship between the levels of the significant explanatory factor (environmental factors at *α* = 0.05) and the different prey taxa. See Table S5 for the abbreviations of the taxa in the diet of *C. zillii*.

The first RDA ordination axis (*F* = 10.2, *p* = 0.002) and all RDA ordination axes (*F* = 4.7, *p* = 0.002) were significant for five populations from two different habitats (Table 5). The first two axes explained 42.29% (RDA1) and 34.45% (RDA2) of the information on the relationship between prey taxa and environmental factors, respectively (Figure 4a). TEMP, DO, and COND are important environmental factors that regulate the changes in prey taxa in different habitats. Among them, COND (*r* ^2^ = 0.66) was the environmental factor with the greatest influence on the food composition, followed by TEMP (*r* ^2^ = 0.51) and DO (*r* ^2^ = 0.35) (Table 5). The first RDA ordination axis (*F* = 7.3, *p* = 0.004) and all RDA ordination axes (*F* = 3.3, *p* = 0.002) were significant for seasonal samples from SKSK. The first two axes explained 28.52% (RDA1) and 24.18% (RDA2) of the information regarding the relationship between prey taxa and environmental factors, respectively (Figure 4b). TN, TD, pH, and PI are the important environmental factors that influence the changes in prey taxa in different seasons of SKSK. Among them, TN (*r* ^2^ = 0.37) was the environmental factor that had the greatest influence on the food composition, followed by TD (*r* ^2^ = 0.33), pH (*r* ^2^ = 0.17) and PI (*r* ^2^ = 0.11) (Table 5).

**TABLE 5:** Results of redundancy analysis (RDA) between diet data and environmental variables for *Coptodon zillii* from five sampling sites and four seasons of SKSK.

Type of grouping	Environmental factors	RDA1	RDA2	*r* ^2^	Pr (> *r*)
Habitat	Water temperature	−0.96	−0.29	0.51	0.0015
	Dissolved oxygen	−0.94	0.34	0.35	0.011
	Conductivity	0.74	0.68	0.66	0.0010
Seasons of SKSK	pH	−0.30	−0.95	0.17	0.0060
	Conductivity	−0.95	−0.32	0.027	0.46
	Turbidity degree	0.59	0.81	0.33	0.0005
	Permanganate index	1.00	0.079	0.11	0.048
	Total phosphorus	−0.17	0.98	0.037	0.34
	Total nitrogen	0.90	−0.44	0.37	0.0005

*Note:* RDA1 and RDA2 represent the first and second ordination axes of RDA analysis, respectively. The values corresponding to RDA1 and RDA2 are the cosine values of the angle between the environmental factor arrow and the ordination axis, indicating the correlation between the environmental factor and the ordination axis.

Abbreviations: Pr, *p*‐value of the significance test of correlation; *r* ^2^, the coefficient of determination of environmental factors on species distribution; The smaller *r* ^2^, the smaller the impact of the environmental factor on species distribution.

There was no significant Mantel correlation between the dietary overlap index and geographic distance between five populations from two different habitat types (*R* ^2^ = 0.0063, *p* = 0.83) (Figure S7). Likewise, there was no significant Mantel correlation between Bray–Curtis distance and geographic distance between populations (*R* ^2^ = 0.37, *p* = 0.064) (Figure S7).

## 4 Discussion

### 4.1 Trophic Plasticity of *C. zillii* Populations

#### 4.1.1 Comparison of Trophic Plasticity in Native and Invasive Populations

Trophic plasticity refers to the adaptability of an organism to reduce potential food competition by altering its diet (Mavraki et al. 2020). It is a form of trophic generalism, meaning that a species is able to feed on a broad food spectrum but only utilizes a portion of it, depending on the environment it inhabits (Riera 2009). Trophic plasticity is considered to be one of the important factors contributing to the successful invasion of exotic fishes (Pettitt‐Wade et al. 2015) and may allow them to colonize different environments, exploit new food resources, and outcompete many native fishes (Cathcart et al. 2019).

Based on previous studies of dietary patterns, our results confirm that *C. zillii* is omnivorous, feeding on a wide variety of foods. Our study showed that the prey taxa of *C. zillii* in the invaded range differed from those in its native range. For example, in its native range, the diet of *C. zillii* in Lake Nasser, Egypt, includes detritus, plant tissues, green algae, diatoms, rotifers, branchiopods, copepods, invertebrates, and others (Shalloof et al. 2020). In the Otamiri River, Nigeria, they feed on algae, vegetative matter, detritus, and aquatic invertebrate larvae (Agbabiaka 2012). In its invasive range, *C. zillii* consumes mainly detritus, algae, macrophytes, and diatoms in the Garmat Ali River, Iraq (Mohamed and Al‐Wan 2020). *C. zillii* from the Arm‐Tigris River in Iraq is herbivorous, feeding on six food categories, including filamentous algae, plant particles and their seeds, organic matter, inorganic sediments, diatoms, and fish eggs (Wahab 2021). The feeding pattern of *C. zillii* from Shadegan wetland in Iran is vegetarian with low animal sources. Its gut contents include macrophytes, fish scales, fish eggs, branchiopods, copepods, and periphyton species (Bavali et al. 2022). In the present study, the highest RRA of phytoplankton (52.84%) followed by detritus (13.49%), zooplankton (12.95%), protozoa (8.79%) and zoobenthos (3.12%), was found in the diet of the five populations from two different habitats. A possible explanation for these different findings is that there are differences in the availability of prey species in different habitats. Another possible explanation is that the DNA metabarcoding technique is more sensitive and can detect a wider range of food categories than traditional morphological identification methods. In the present study, a total of 22 phyla of food categories were identified by the DNA metabarcoding technique in the stomach contents of redbelly tilapia samples from two rivers and three reservoirs, of which 16 taxa have not been reported in previous traditional morphological identification of stomach contents, including Ascomycota, Ciliophora, Bryozoa, Microsporidia, Streptophyta, Oomycota, Cnidaria, Cercozoa, Chytridiomycota, Basidiomycota, Cryptomycota, Nemertea, Picozoa, Gastrotricha, Apicomplexa, and Mollusca. Therefore, to facilitate the comparison of the degree of trophic plasticity in the native and invasive ranges, we used the prey species and proportions of *C. zillii* reported in the literature (Agbabiaka 2012; Wahab 2021; Mohamed and Al‐Wan 2020; Shalloof et al. 2020) to characterize the niche breadth index (*B*) of different populations. In its native range, the *B* of the population from Nasser Lake in Egypt and Otamiri River in Nigeria was 5.16 and 8.32, respectively. In its invasive range, the *B* of the population from the Arm‐Tigris River in Iraq, Garmat Ali River, and Shadegan Wetland in Iran was 2.36, 3.38, and 3.78, respectively. In the present study, the *B* of the populations from the two river habitats was 1.37 (NDJ) and 2.28 (DJ), and the *B* of the populations from the three reservoir habitats was 1.94 (QDH), 2.03 (SKSK), and 4.25 (XJ), respectively. The results of the above studies showed that the *B* of the native populations was all greater than those of the invasive populations. Previous studies have shown that the *B* of species is influenced by a variety of abiotic and biotic factors, such as resource density and diversity, population densities, competitors, and predators (Olsson et al. 2009). Moreover, ecological niche contraction usually occurs with increased interspecific competition (Bolnick et al. 2010). We suggest that invasive populations may use only the best food resources by reducing their spatial ecological niche in response to environmental pressures such as interspecific competition and predation in the new habitat, as compared to the native populations (MacArthur and Pianka 1966). On the other side, as a versatile predator with a strong sense of territoriality, *C. zillii* may also reduce intraspecific competition by reducing overlap in resource use (Bolnick et al. 2007). All of these factors may contribute to the contraction of the trophic ecological niche of invasive populations. Limited evidence suggests that species can rapidly change their trophic ecological niche once they enter a new environment (Comte et al. 2016). For example, Tran et al. (2015) found that the invasion of topmouth gudgeon (*Pseudorasbora parva*) led to a differentiation of trophic ecological niches, in part because they reduce their ecological niche width when they coexist with other species, thereby facilitating their coexistence in the invaded ecosystem.

#### 4.1.2 Spatial Variation in the Trophic Plasticity of Invasive Populations

Geographic and ecological habitats can play an active role in the feeding strategies of fish by providing different food supplies (Garcia et al. 2018). Studies have shown that some invasive freshwater fishes are able to flexibly adapt their feeding strategies to prey availability under different habitat conditions (i.e., rivers vs. reservoirs) with a wide range of dietary plasticity (Marchetti et al. 2004). For example, trophic plasticity facilitates the invasion of bleak ( *Alburnus alburnus* ) in a variety of lentic (reservoirs) and lotic (rivers) habitats in the Iberian Peninsula (Almeida et al. 2017). The Eastern mosquitofish ( *Gambusia holbrooki* ) that invaded northwestern Turkey showed a generalized feeding strategy in lotic and lentic habitats (Saç 2023). In our study, the RRA of different categories of food in the diet of *C. zillii* populations in different habitats varied considerably. For example, the RRA of phytoplankton in the diet of the NDJ population was 85.89%, which was much higher than those of other populations. The RRA of protozoa in the diet of the XJ and SKSK populations reached 17.47% and 15.16%, respectively, which was much higher than those of other populations. The RRA of zooplankton in the diet of the DJ population was 43.79%, which was much higher than those of other populations. The RRA of zoobenthos in the diet of the SKSK population was 10.95%, which was much higher than those of other populations, and no zoobenthos was detected in the diet of the NDJ population. The RRA of detritus in the diet of the QDH and XJ populations was 28.07% and 26.11%, respectively, which were much higher than those of the other populations. In riverine habitats (DJ and NDJ), *C. zillii* preferred phytoplankton and zooplankton followed by detritus and protozoa. In reservoir habitats, phytoplankton was the most consumed food, followed by detritus, protozoa, and zoobenthos. These results suggest that *C. zillii* consumed different food resources in different habitats and showed a wide range of plasticity in dietary traits.

In general, river ecosystems are structurally more complex than reservoirs. Consequently, the nutrient resources that rivers can provide are usually more diverse (Terra and Arau'jo 2011). Therefore, we expected a higher level of trophic diversity in river populations, and the results of this study largely support this hypothesis. In this study, the Shannon‐Wiener index (*H*′) was used to characterize the food diversity of river and reservoir populations. According to Encina et al. (2004), low values indicate diets dominated by a small number of prey (specialist predators) and high values indicate extensive diets. Diets with values greater than 2 were considered high, while values less than 1 were considered low. We found that the highest *H*′ was detected in the NDJ population, followed by the XJ population. The *H*′ of the river populations (NDJ and DJ) was higher than those of the two reservoir populations (QDH and SKSK). The H′ of the XJ population was higher than that of the DJ population, but the difference between them was not significant (*p* > 0.05). Meanwhile, the *H*′ of two river populations and three reservoir populations was higher than 2 (*H*′ = 2.50–4.85), indicating that *C. zillii* has a wide range of food spectrum in both river and reservoir habitats in China, and the trophic diversity level of the river populations was higher than that of the reservoir populations.

#### 4.1.3 Seasonal Changes in the Trophic Plasticity of Invasive Populations

Assessing seasonal trophic variation in invasive fish populations is critical because such differences may result in different impacts on invasive ecosystems throughout the year. Changes in seasonal trophic ecological niches of invasive fish populations can reflect changes in diet (e.g., utilization of more diverse prey). Changes in the size of trophic ecological niche space reflect the response of available resources to environmental drivers; for example, differences in prey availability across seasons may result in seasonal shifts in ecological niches (Haubrock et al. 2021; Hedden et al. 2022). For example, seasonal variation in the diet of channel catfish ( *Ictalurus punctatus* ) has been reported in its native (Holland and Peters 1992) and introduced (Hedden et al. 2022) ranges, with a wider range of food items in spring. In the present study, the RRA of different food categories in the diet of the *C. zillii* population varied considerably between seasons. For example, the RRA of phytoplankton was highest in spring (24.92%) and lowest in fall (3.22%). The RRA of protozoa and zooplankton was highest in fall at 47.95% and 17.07%, respectively. The RRA of protozoa and zoobenthos was lowest in spring at 17.79% and 12.18%, respectively. The RRA of zoobenthos was highest in summer (34.81%). These results suggest that *C. zillii* consumed different food resources in different seasons and showed a wide range of plasticity in dietary traits.

Water temperature in reservoirs changes seasonally, and seasonal changes in water temperature are one of the main factors affecting the seasonal succession of food resources (including phytoplankton, zooplankton, protozoa, etc.) for invasive fishes. Summer is the season with the highest average water temperatures in SKSK, and therefore, the nutrient resources that can be provided are usually more diverse. We predicted that the highest level of trophic diversity in SKSK would occur during the summer months, and the results of the present study largely supported this hypothesis. We found that the *H*′ and *B* of summer were higher than those of the other seasons, and the *B* of winter was the lowest (2.65). The *H*′ of the population samples in different seasons ranged from 3.81 to 4.22, which was higher than 2. This indicated that the population in SKSK had a wide range of food spectrum in different seasons and the level of trophic diversity in summer was higher than those in other seasons.

### 4.2 Dietary Overlap and Potential Impacts Between *C. zillii* and Native Omnivorous Fishes

Species invasions alter interactions within and between communities, with potentially serious consequences for biodiversity and ecosystems (Pimm et al. 1991). A high degree of trophic plasticity allows invasive species to adjust their feeding behavior when ecological niches overlap with native species (Zengeya et al. 2011). Invasions of generalist predators often result in reduced abundances of native species (David et al. 2017). The global invasion of carp ( *Cyprinus carpio* ) is facilitated by the high trophic plasticity, which often dominates aquatic ecosystems and threatens native aquatic taxa (e.g., fish, aquatic plants, etc.) through both top‐down and bottom‐up processes (e.g., predation and alteration of trophic levels and turbidity) (Weber and Brown 2009). The invasion of rainbow trout ( *Oncorhynchus mykiss* ) may lead to changes in ecological niches and declines in native fish populations through predation or competitive exclusion of food resources (Shelton et al. 2015). The invasion of *O. niloticus* and *C. rendalli* altered the structure of tropical freshwater food webs in artificial reservoirs (Lake Gatun and Lake Bayano) in central Panama (Sharpe et al. 2023).

In our study, *C. zillii* was omnivorous, with a wide range of food spectrum and a high degree of trophic plasticity, and it was able to adjust its trophic position in response to food availability. We predicted that the invasion of *C. zillii* could change the structure and dynamics of food webs through various mechanisms (e.g., predation and competition), thereby affecting the dietary and trophic ecological position of native fishes. On the one hand, *C. zillii* may alter the energy supply of other fishes by feeding at the bottom of the food web (feed on phytoplankton), monopolizing and retaining sufficient basal food resources, even if they do not directly compete with them for food. This has been identified as one of the main invasion mechanisms for common carp ( *C. carpio* ), which also limit native fish biomass by monopolizing and retaining basal food resources in Australian rivers (Marshall et al. 2019). Nutrition of common fishes (e.g., *Pelteobagrus fulvidraco* and *Pseudobagrus nitidus*) in QDH and SKSK is highly dependent on algal‐feeding macroinvertebrates (Hu et al. 2019), and thus algal feeding by *C. zillii* may limit the food source for herbivorous invertebrates, thereby limiting the quantity and quality of food for native fish communities. On the other hand, omnivorous freshwater fishes are widespread in the food web of rivers and reservoirs in southern China, and when there is a dietary overlap between *C. zillii* and native fishes, *C. zillii* may pose a competitive threat to native omnivorous fishes in order to access limited food resources. Based on the published literature, we organized the diets of three common omnivorous fishes in southern China [ *Xenocypris davidi* (Xu 1988), *Xenocypris argentea* (Xu and Liao 1984), and Prussian carp (*Carassius auratus gibelio*) (Zhang et al. 2020)], where *X. davidi* is indigenous to DJ, NDJ, and QDH, *X. argentea* is indigenous to SKSK, DJ, and QDH, and Prussian carp is indigenous to SKSK and QDH. We calculated the dietary overlap indices of *C. zillii* and these indigenous fish species according to the formula of Schoener (1970) and found that the dietary overlap indices (Schoener overlap index) of *C. zillii* with *X. davidi* , *X. argentea* , and *Carassius auratus gibelio* were 0.22, 0.28, and 0.43, respectively. Although none of the overlap indices exceeded the threshold value (0.6), this already suggests that there is a partial dietary overlap between *C. zillii* and these native fishes. It is noteworthy that the proportion of phytoplankton in the diets of *C. zillii* and Prussian carp was very close to each other, which may have led to a high degree of ecological niche overlap between these two species. It can be hypothesized that in SKSK and QDH, *C. zillii* did not take advantage of vacant trophic ecological niches in these two aquatic ecosystems but rather utilized similar food resources as Prussian carp to potentially displace it competitively.

### 4.3 Management Implications of *Coptodon zillii*

For redbelly tilapia, prevention and early detection can prevent the introduction and establishment of its population, but when its invasion enters the stage of spread and outbreak, control and eradication techniques are needed to control its population density. We believe that detailed prevention and control recommendations for redbelly tilapia include the following two aspects.

#### 4.3.1 Eradication, Containment, and Suppression

For water systems with a small population of redbelly tilapia, which are the occurrence points of the dispersal front, efficient physical clearance, chemical control, and other measures are adopted to carry out early eradication and extinction. Electric fishing and gill net trapping are also effective eradication methods in areas where redbelly tilapia occurs locally. It is necessary to adopt a comprehensive management approach that combines physical control, chemical control, and biological control measures to effectively suppress the rapid spread of the redbelly tilapia in a large distribution area.

##### 4.3.1.1 Physical Prevention and Control

Regularly carry out centralized fishing and clearance in waters with a high population density of redbelly tilapia to reduce its population size. At the same time, during the breeding season of redbelly tilapia, increase the fishing intensity in the waters where it lays eggs and control the number of its seedlings.

##### 4.3.1.2 Chemical Control

In response to the characteristics of small size and high density of redbelly tilapia, a toxic bait containing attractants can be developed to lure redbelly tilapia into specialized nets for poisoning.

##### 4.3.1.3 Biological Control

On the one hand, during the breeding season of redbelly tilapia, specific sex pheromones are used to trap sexually mature individuals (parent fish), effectively controlling the size of the breeding population. On the other hand, in waters with a large population size of redbelly tilapia, indigenous carnivorous fish are regularly released to suppress the number of eggs, fry, and juveniles of redbelly tilapia through predation and competition, thereby reducing their population density.

#### 4.3.2 Maintain the Ecological Balance of Local Water Bodies and Protect Aquatic Biodiversity

In order to effectively stop the invasion of *C. zillii*, we believe that it is extremely important to maintain the ecological balance of local water bodies and protect aquatic biodiversity. According to the Diversity Resistant Hypothesis (DRH) (Elton 1958), ecosystems with high species diversity are more resistant to invasive alien species than those with low diversity, and there is a negative correlation between diversity and invasiveness. Recent studies have shown that local fish can alleviate the invasion of alien fish through resource competition, habitat limitations, and direct predation (Gu, Jia, et al. 2023; Gu, Luo, et al. 2023). We believe that from the perspective of protecting and restoring aquatic ecosystems, the following two measures can be taken to prevent further invasion of redbelly tilapia. Firstly, accelerate the construction of the legal system, formulate and improve laws and regulations on aquatic ecological environment protection. Secondly, restore degraded aquatic ecosystems, gradually restore aquatic ecological functions, maintain the health of aquatic ecosystems, and protect aquatic biodiversity.

## 5 Conclusions

For the first time, we made a direct comparison of the dietary patterns of Chinese invasive populations of *C. zillii* at a large geographic scale. The results revealed that *C. zillii* is an omnivorous fish that exhibits generalized feeding strategies in both river and reservoir habitats. In the aquatic ecosystems of southern China, populations of *C. zillii* showed high trophic plasticity in different spaces (rivers or reservoirs) and at different times (seasons). Aquatic environmental factors were key drivers of dietary differences between populations in different habitats and between populations in different seasons. We did not find significant correlations between individual standard length and dietary patterns. Although the existence of adverse effects of *C. zillii* on the structure and dynamics of the native food web has not been confirmed, we predict that *C. zillii* will pose a potential threat to the dietary and trophic ecological niches of native omnivorous fishes.

## Author Contributions

**Shoujie Tang:** conceptualization (lead), data curation (lead), methodology (lead), writing – original draft (lead). **Ying Xing:** data curation (equal), methodology (equal). **Temesgen Tola Geletu:** writing – review and editing (equal). **Jinliang Zhao:** funding acquisition (lead), supervision (lead).

## Ethics Statement

The experimental protocol was approved by the Institutional Animal Care and Use Committee (IACUC) of Shanghai Ocean University (permit number: SHOUDW2024085) and complies with the Guidelines on Ethical Treatment of Experimental Animals established by the Ministry of Science and Technology, China.

## Conflicts of Interest

The authors declare no conflicts of interest.

## Supporting information


Data S1.

## Data Availability

Data are available via ZENODO https://doi.org/10.5281/zenodo.14089210.
