# Supplementary material for: Trophic Plasticity of the Invasive Redbelly Tilapia (Coptodon zillii) in China Inferred From DNA Metabarcoding Analysis
Source: Ecol Evol. 2025 Apr 3;15(4):e71118. doi: 10.1002/ece3.71118 (PMC11968421; doi:10.1002/ece3.71118)
Supplement: Supplementary file 1 — Data S1. [file ECE3-15-e71118-s001.docx]

Supplementary figures


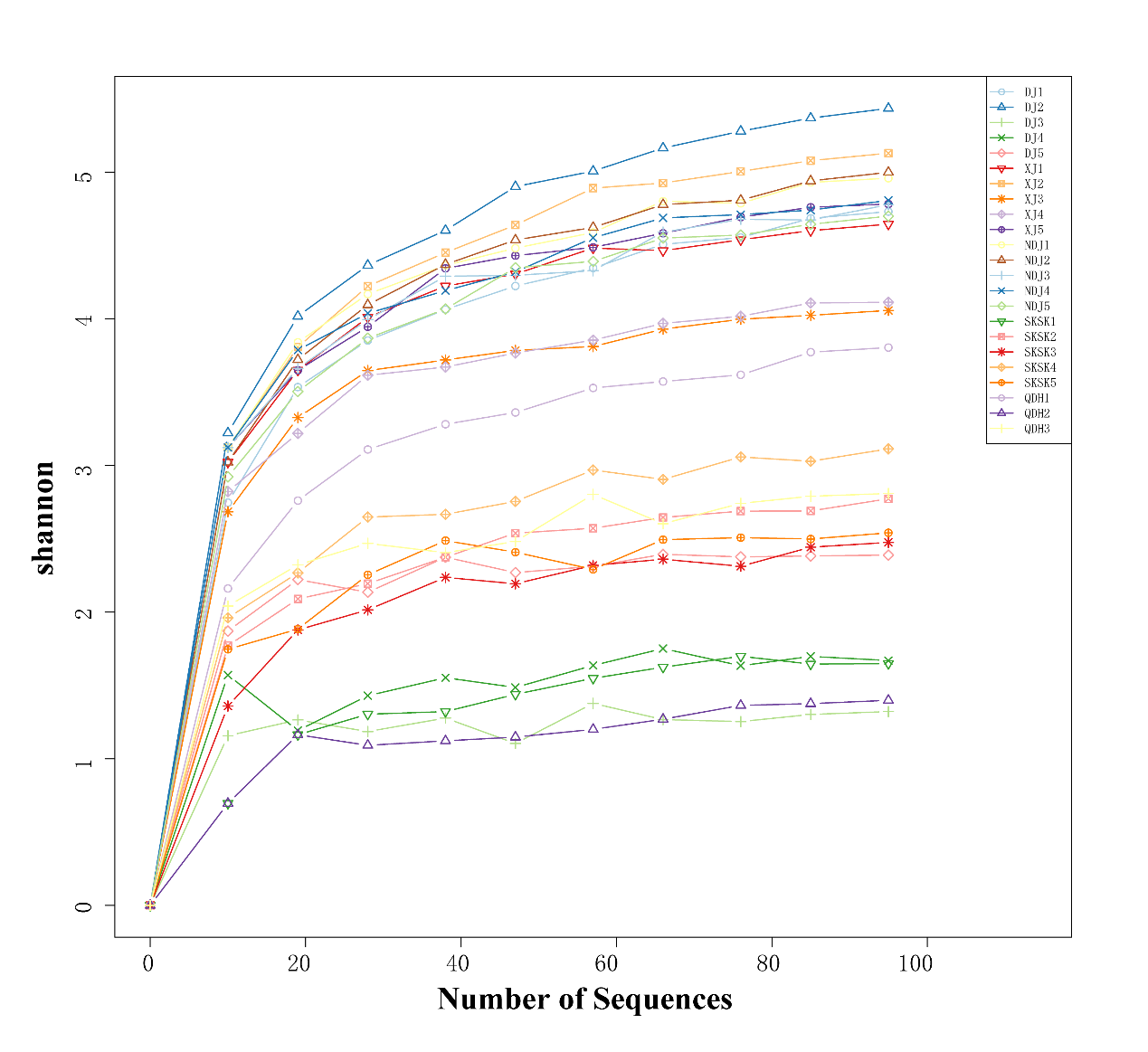


(**a**)


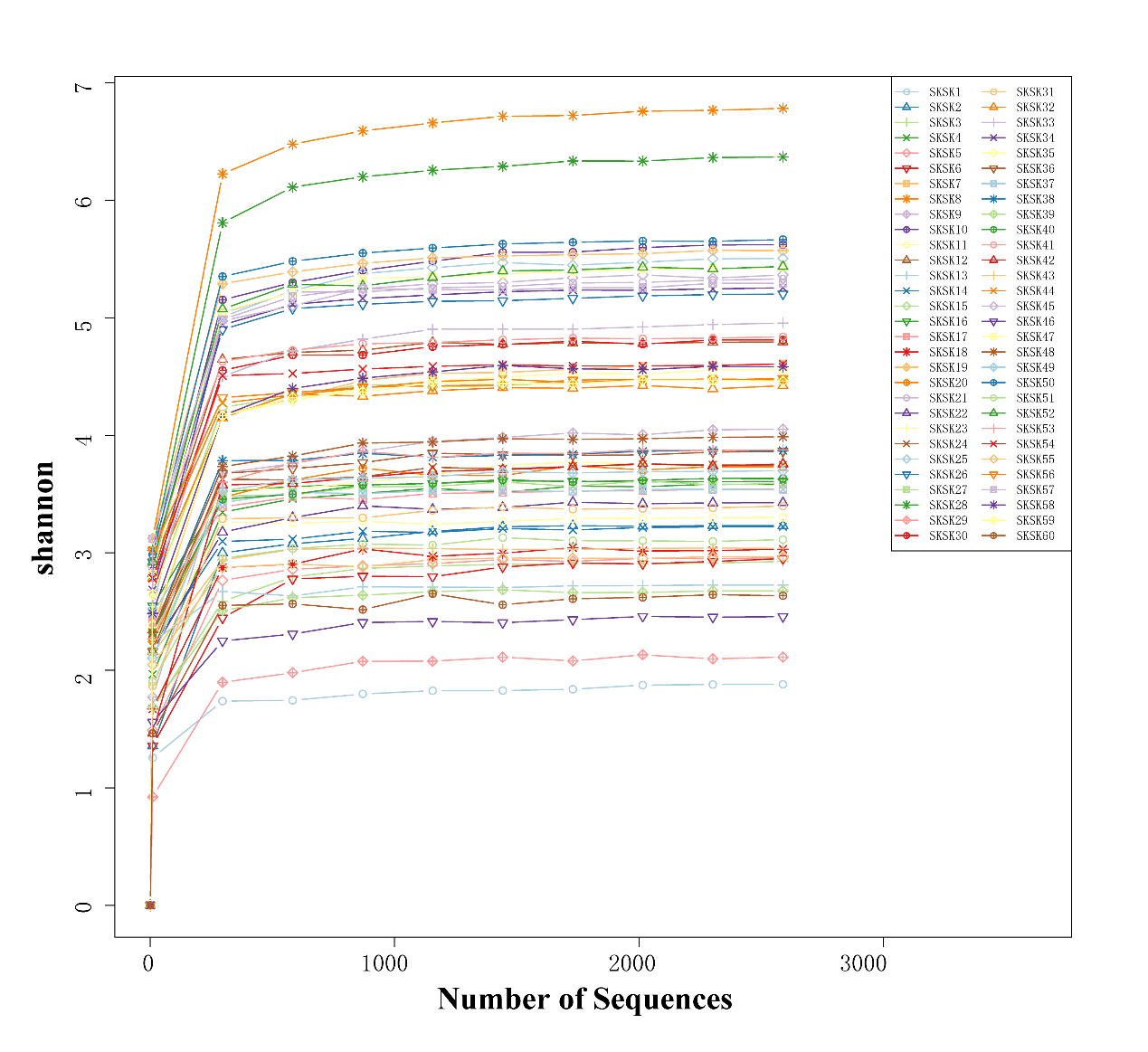


(**b**)

**Fig. S1** Rarefaction curves to evaluate the completeness of the sequencing effort at describing the diversity of dietary items in the stomach contents of *C. zillii* from five sampling locations (**a**) and in four seasons of SKSK (**b**). The horizontal axis denotes the amount of sequencing data for each sample, and the vertical axis denotes the Shannon-Wiener index. When the curve tends to be flat, it indicates that the amount of sequencing data is leveling off, and more data will not have a significant impact on the diversity index. The sample ID for five sampling locations and four seasons (spring, summer, autumn, and winter) of SKSK are shown in Table S4.


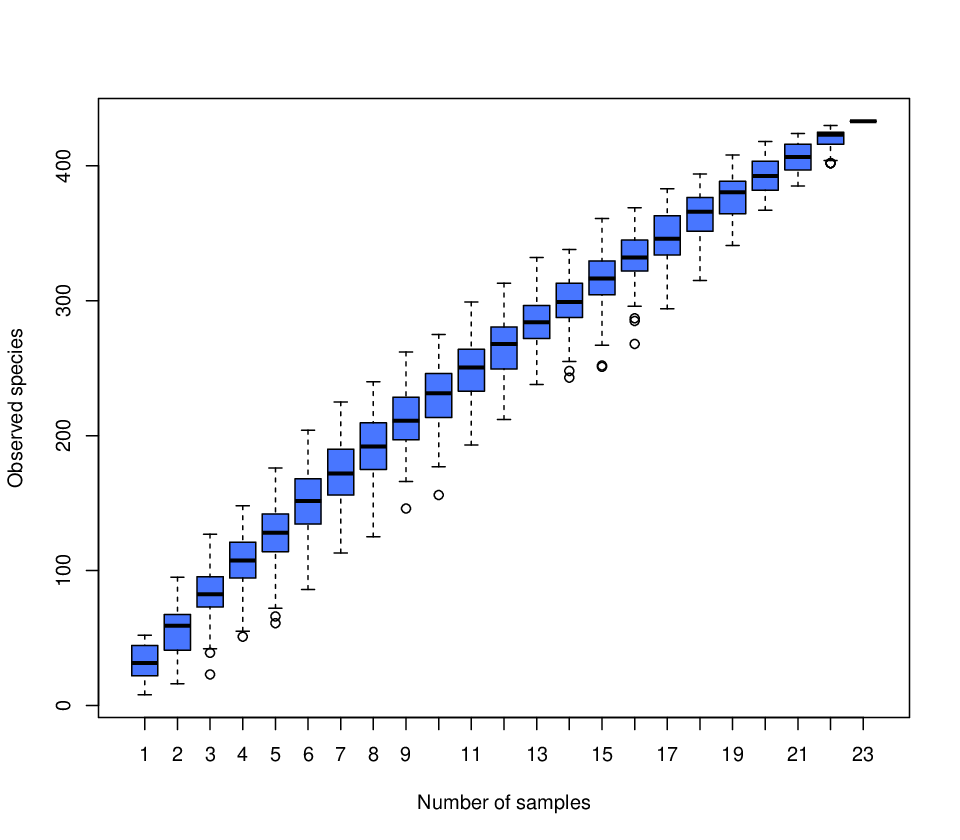

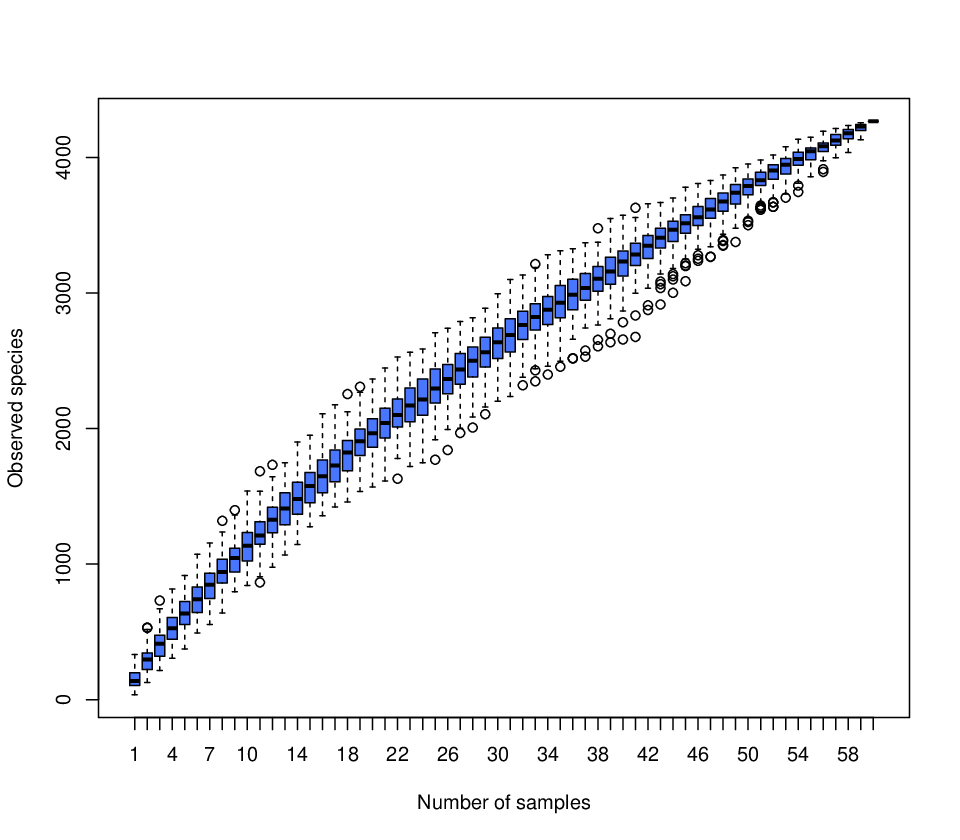


(**a**) (**b**)

**Fig. S2** Species Accumulation Boxplot for each sample from five sampling locations (**a**) and in four seasons of SKSK (**b**). The horizontal axis represents the sample size, and the vertical axis represents the number of sequences after sampling.


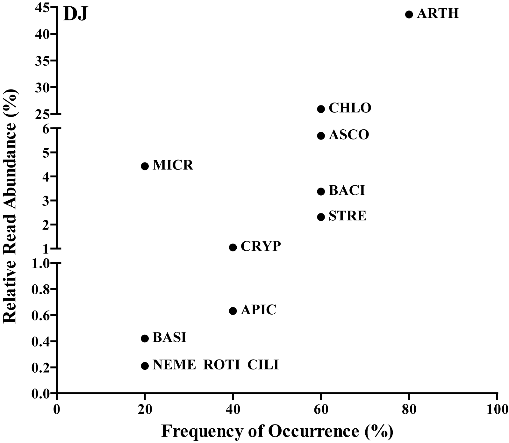

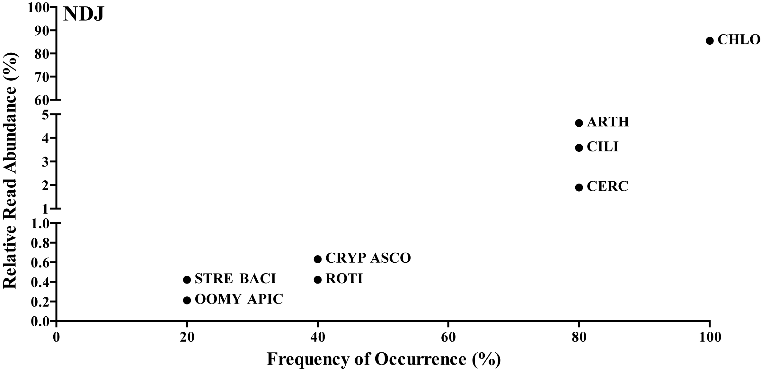


(**a**) **(b)**


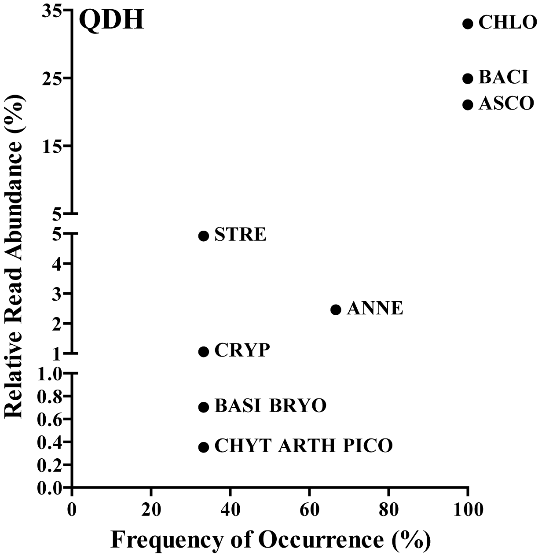

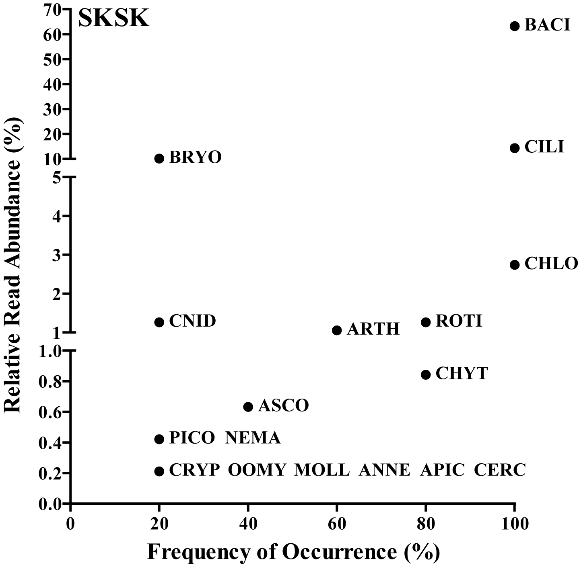


**(c) (d)**


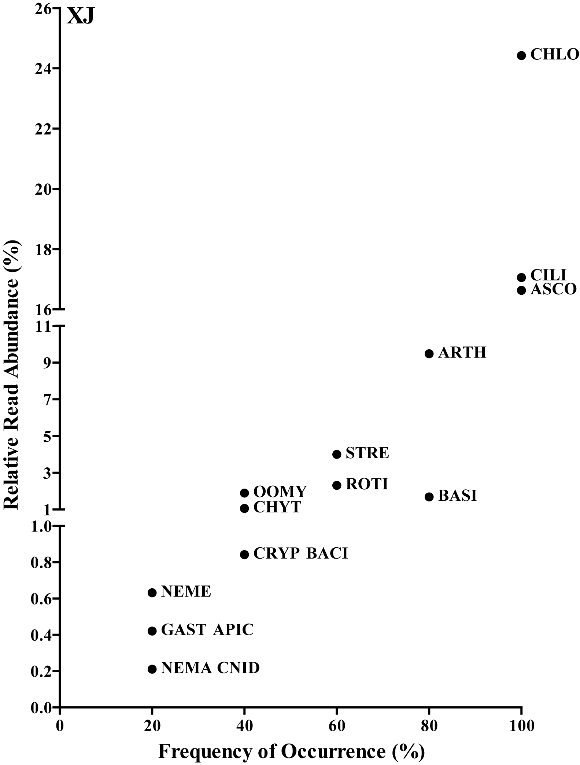


**(e)**

**Fig. S3** Modified Costello (1990) feeding strategy diagram for *C. zillii* in five sampling locations: (**a**) DJ, (**b**) NDJ, (**c**) QDH, (**d**) SKSK, (**e**) XJ. Relative read abundance (RRA) plotted against the frequency of occurrence (FO) of food items in the diet of the species.


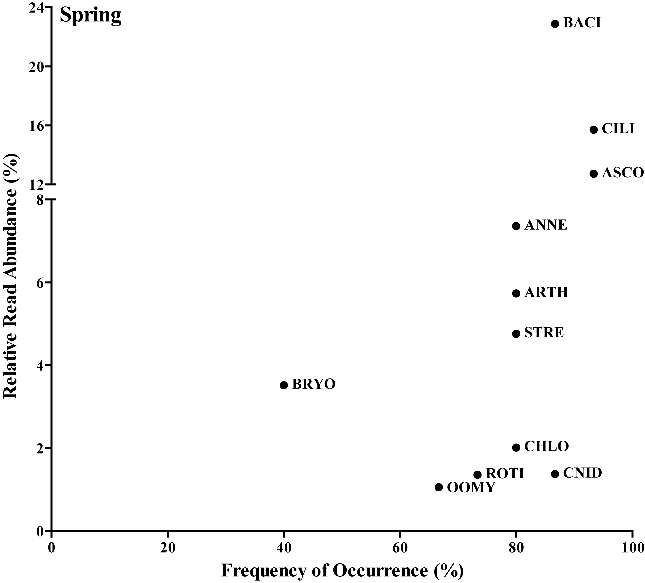

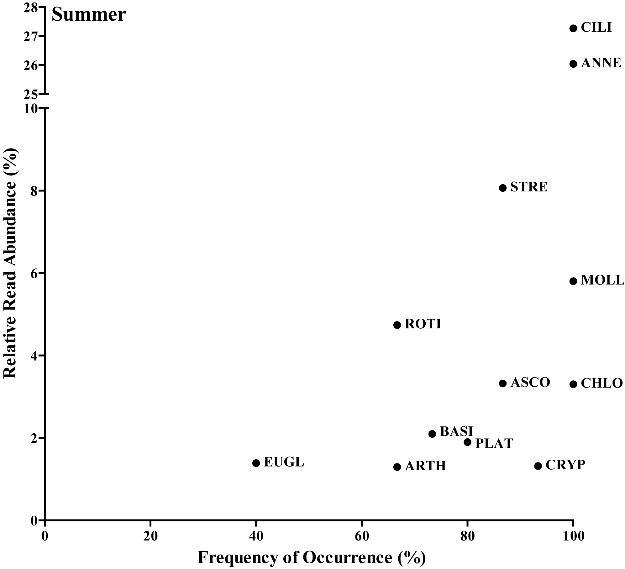


**(a) (b)**


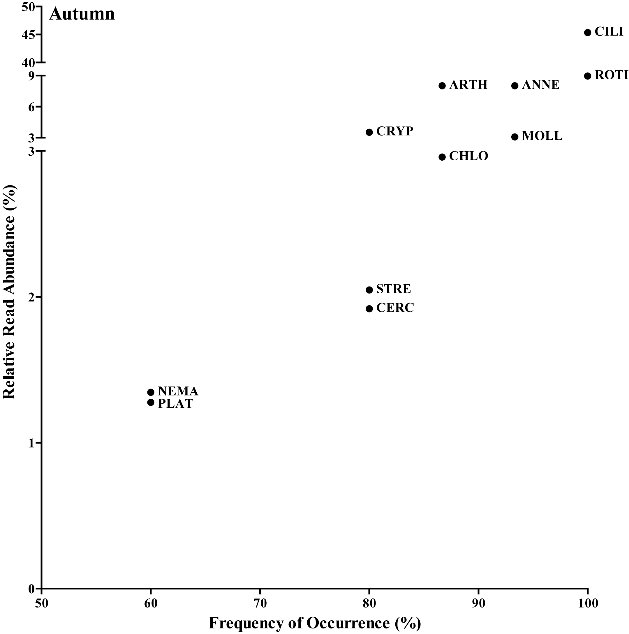

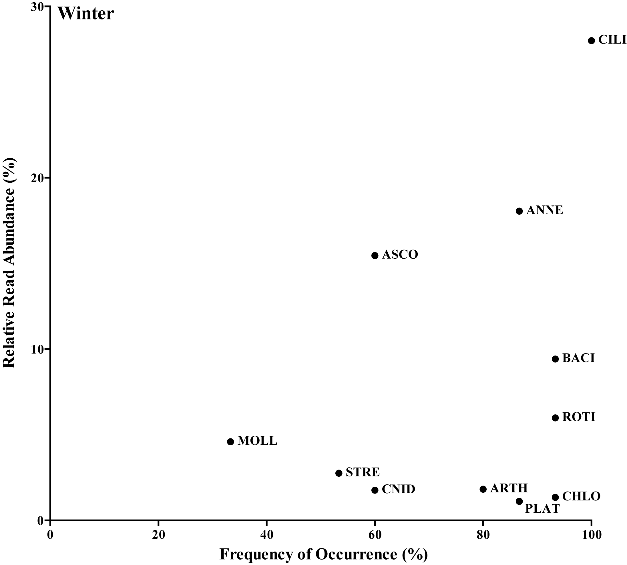


**(c) (d)**

**Fig. S4** Modified Costello (1990) feeding strategy diagram for *C. zillii* in four seasons of SKSK: (**a**) Spring, (**b**) Summer, (**c**) Autumn, (**d**) Winter. Relative read abundance (RRA>1%) plotted against the frequency of occurrence (FO) of food items in the diet of the species.


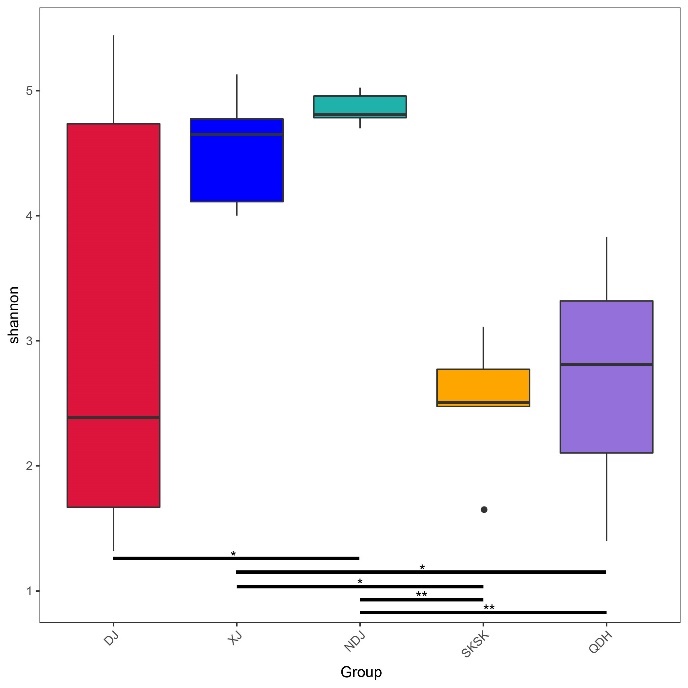

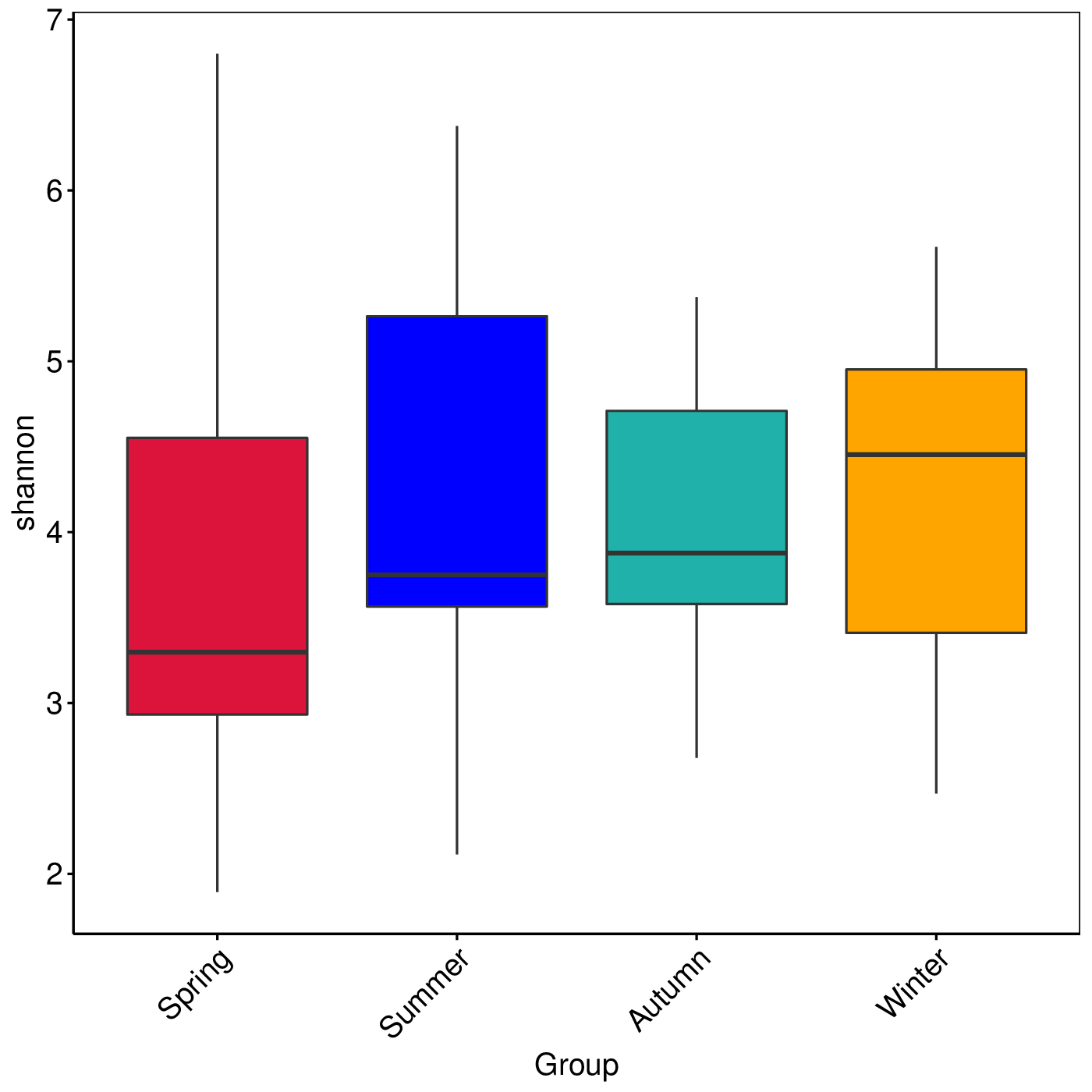


(**a**) (**b**)


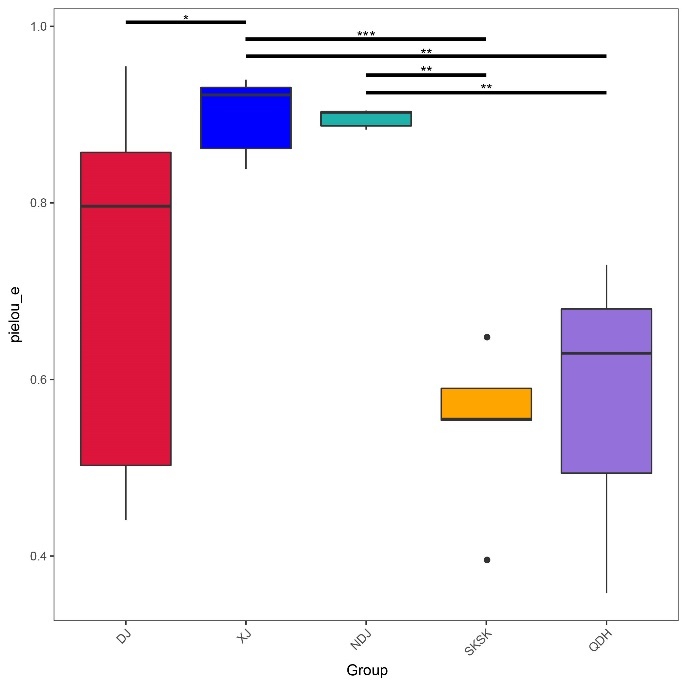

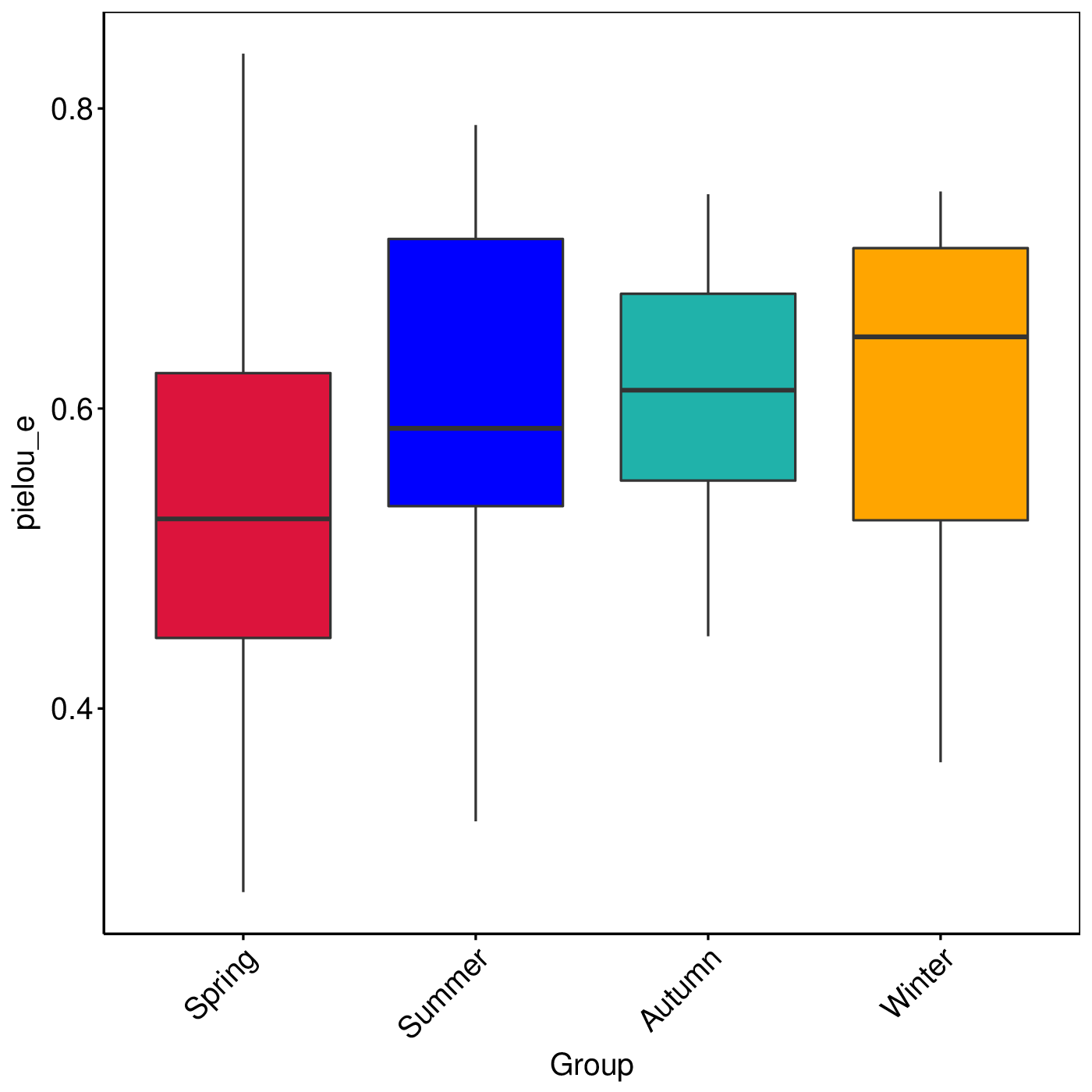


(**c**) (**d**)

**Fig. S5** Box-plot of Shannon-Wiener index for *C. zillii* in five sampling locations (**a**) and four seasons of SKSK (**b**). Box-plot of Pielou’s evenness index for *C. zillii* in five sampling locations (**c**) and four seasons of SKSK (**d**). In each panel, the abscissa is the group, and the ordinate is the value of Shannon-Wiener index (shannon) and Pielou’s evenness index (pielou_e), respectively.


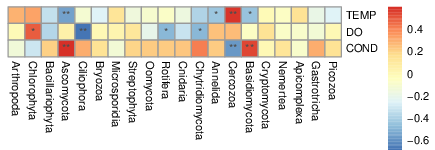


(**a**)


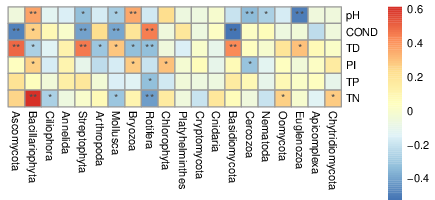


(**b**)

**Fig. S6** Heatmap of Spearman correlation analysis between prey items and environmental factors for *C. zillii* among five sampling locations (**a**) and among four seasons of SKSK (**b**). The vertical axis represents environmental factor information. The horizontal axis represents species information. And the value corresponding to the heatmap is the Spearman correlation coefficient *r*, which is between -1 and 1, *r*<0 indicates a negative correlation, *r*> 0 indicates a positive correlation, and ^*^ denotes *P*<0.05, ^**^ denotes *P*<0.01.


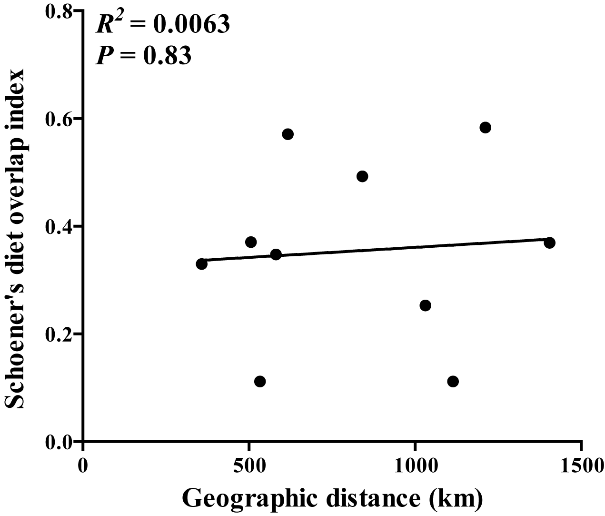

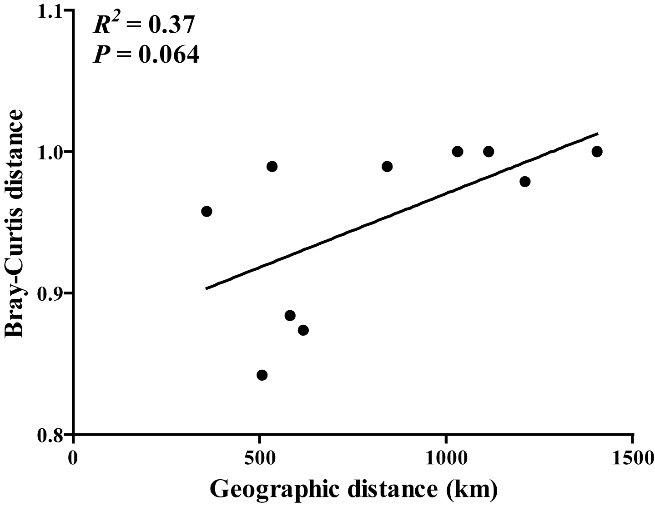


(**a**) (**b**)

**Fig. S7** Correlation between geographic distances and dietary differences between populations for *C. zillii*. (**a**) Correlation between geographic distances and dietary overlap index between populations. The slope of the linear regression is (3.74e-05) ± (1.66e-04), and the intercept is 0.32 ± 0.15. The correlation (*y* = 3.74e-05*x* + 0.32, *R*^2^ = 0.0063) was not significant (*P* = 0.83). (**b**) Correlation between geographic distances and Bray-Curtis distance between populations. The slope of the linear regression is (1.044e-04) ± (4.85e-05), and the intercept is 0.87 ± 0.043. The correlation (*y* = 1.044e-04*x* + 0.87, *R*^2^ = 0.37) was not significant (*P* = 0.064). Values of Mantel’s correlation test are provided at the top of the panel.

Supplementary tables

**Table S1** List of the 5 sampling sites from which specimens of *Coptodon zillii* were collected including their location, number of fish caught (F: females; M: males), environmental parameters (Dissolved oxygen, pH, Conductivity, Water temperature, Turbidity degree, Permanganate index, Ammonia nitrogen, Total phosphorus, Total nitrogen) downloaded from China National Environmental Monitoring Centre (<https://www.cnemc.cn/>), and site-specific climate data (Daily mean temperature, Daily maximum and minimum temperature, and Daily precipitation sum) downloaded from Public Meteorological Service Center of China Meteorological Administration (https://www.tianqi24.com/) at 0.1 degrees resolution

| Population | Location | Habitat type | Sampling date | Latitude (N) | Longitude  (E) | No. of samples (F\M) | Water temperature  (℃) | pH | Dissolved oxygen (mg/L) | Conductivity (μS/cm) | Turbidity degree  (NTU) | Permanganate index (mg/L) | Ammonia nitrogen (mg/L) | Total phosphorus  (mg/L) | Total nitrogen (mg/L) | Daily mean temperature  (℃) | Daily minimum temperature(℃) | Daily maximum temperature(℃) | Daily precipitation sum (mm) |
| --- | --- | --- | --- | --- | --- | --- | --- | --- | --- | --- | --- | --- | --- | --- | --- | --- | --- | --- | --- |
| DJ | Huizhou City, Guangdong Province | River | 26/10/2022 | 23.16^o^N | 114.60^o^E | 51(26\25) | 24.31 | 7.19 | 7.71 | 111.01 | 20.37 | 1.77 | 0.044 | 0.044 | 1.72 | 23.5 | 19 | 28 | 466 |
| NDJ | Ding'an County, Hainan Province | River | 3/11/2022 | 19.72^o^N | 110.37^o^E | 55(23\32) | 27.26 | 7.43 | 7.89 | 101.33 | 29.89 | 2.68 | 0.047 | 0.086 | 1.28 | 25.5 | 21 | 30 | 1076.1 |
| QDH | Chun'an County, Zhejiang Province | Reservoir | 15/10/2022 | 29.58^o^N | 119.08^o^E | 110(59\51) | 21.32 | 8.68 | 9.11 | 139.11 | 4.04 | 1.63 | 0.040 | 0.012 | 0.94 | 18.5 | 14 | 23 | 1125.7 |
| SKSK | Nanping City, Fujian Province | Reservoir | 22/10/2022 | 26.40^o^N | 118.50^o^E | 63(33\30) | 23.83 | 6.99 | 6.61 | 118.64 | 27.20 | 1.89 | 0.099 | 0.065 | 1.70 | 20.5 | 15 | 26 | 1403.1 |
| XJ | Xincheng County, Guangxi Zhuang Autonomous Region | Reservoir | 14/11/2022 | 23.97^o^N | 108.61^o^E | 68(31\37) | 22.24 | 7.78 | 7.15 | 362.69 | 7.35 | 0.70 | 0.025 | 0.0073 | 1.80 | 21.0 | 17 | 25 | 709.5 |

**Table S2** Description of stomach fullness degrees used in this study

| Degree | Level of fullness | Description |
| --- | --- | --- |
| 0 | Empty stomach | No trace of food or watery digested |
| 1 | ¼ full stomach | Only a little food item was found in this stomach |
| 2 | ½ full stomach | Stomach was filled with food items to approximately half of its length |
| 3 | ¾ full stomach | Stomach was nearly filled with food item |
| 4 | Full stomach | Stomach was completely filled with food items and appeared cylindrical |
| 5 | Swollen stomach | Stomach was extremely packed with food items and appeared swollen |

**Table S3** Morphological data of eighty-three specimens of *C. zillii* for diet analysis between habitat types and seasons

| Character |  | Habitat type | | | | | Seasons of SKSK | | | |
| --- | --- | --- | --- | --- | --- | --- | --- | --- | --- | --- |
|  |  | River | | Reservoir | | | Spring | Summer | Autumn | Winter |
|  |  | DJ | NDJ | QDH | SKSK | XJ |  |  |  |  |
| Standard length | (mean ± SE) (mm) | 111.4±3.65 | 145.5±1.89 | 111.9±1.043 | 113.3±1.92 | 105.4±1.42 | 141.7±9.30 | 121.3±6.65 | 104.8±4.75 | 125.3±5.75 |
|  | range (mm) | 69.0～166.0 | 121.0～173.0 | 91.0～146.0 | 91.0～160.0 | 84.0～128.0 | 82.0～220.0 | 92.0～163.0 | 76.0～138.0 | 103.0～180.0 |
| Body weight | (mean± SE) (g) | 54.47±5.23 | 105.8±4.43 | 56.63±1.62 | 58.62±3.30 | 44.8±1.80 | 154.8±26.25 | 80.73±14.09 | 43.93±5.75 | 82.60±14.04 |
|  | range (g) | 12.08～156.70 | 55.75～188.40 | 33.67～110.10 | 27.02～161.7 | 21.42～74.83 | 49.56～463.2 | 27.21～187.3 | 16.20～85.93 | 38.44～208.8 |

SE represents standard error.

**Table S4** Barcode sequences for each sample used in this study

|  |  | Sample ID | Barcode sequence (5’-3’) connected to the left end of the primer 528F | Barcode sequence (5’-3’) connected to the right end of the primer 706R |
| --- | --- | --- | --- | --- |
| Habitat type | River | DJ1 | CAAGACTA | ATAGCGAC |
|  |  | DJ2 | CAAGACTA | CCTCTATC |
|  |  | DJ3 | CAAGACTA | CGGATTGC |
|  |  | DJ4 | CAAGACTA | GAGTTAGC |
|  |  | DJ5 | CAAGACTA | GATGAATC |
|  |  | NDJ1 | GCCACATA | AATGTTGC |
|  |  | NDJ2 | GCCACATA | AGCACCTC |
|  |  | NDJ3 | GCCACATA | ATAGCGAC |
|  |  | NDJ4 | GCCACATA | CCTCTATC |
|  |  | NDJ5 | GCCACATA | CGGATTGC |
|  | Reservoir | QDH1 | CGAACTTA | AATGTTGC |
|  |  | QDH2 | CGAACTTA | AGCACCTC |
|  |  | QDH3 | CGAACTTA | ATAGCGAC |
|  |  | SKSK1 | AACGTGAT | AATCCGTC |
|  |  | SKSK2 | AACGTGAT | AATGTTGC |
|  |  | SKSK3 | AACGTGAT | AGCACCTC |
|  |  | SKSK4 | AACGTGAT | ATAGCGAC |
|  |  | SKSK5 | AACGTGAT | CCTCTATC |
|  |  | XJ1 | GCCACATA | GGTGCGAA |
|  |  | XJ2 | GCCACATA | GTCGTAGA |
|  |  | XJ3 | GCCACATA | TCTTCACA |
|  |  | XJ4 | GCCACATA | TTCACGCA |
|  |  | XJ5 | GCCACATA | AATCCGTC |
| Seasons of SKSK | Spring | SKSK1 | AACGTGAT | AATCCGTC |
|  |  | SKSK2 | AACGTGAT | AATGTTGC |
|  |  | SKSK3 | AACGTGAT | AGCACCTC |
|  |  | SKSK4 | AACGTGAT | ATAGCGAC |
|  |  | SKSK5 | AACGTGAT | CCTCTATC |
|  |  | SKSK6 | CGAACTTA | AATCCGTC |
|  |  | SKSK7 | CGAACTTA | AATGTTGC |
|  |  | SKSK8 | CGAACTTA | AGCACCTC |
|  |  | SKSK9 | CGAACTTA | ATAGCGAC |
|  |  | SKSK10 | CGAACTTA | CCTCTATC |
|  |  | SKSK11 | CTGGCATA | TCTTCACA |
|  |  | SKSK12 | CTGGCATA | TTCACGCA |
|  |  | SKSK13 | CTGGCATA | AATCCGTC |
|  |  | SKSK14 | CTGGCATA | AATGTTGC |
|  |  | SKSK15 | ACTTAGCC | GAGCTACA |
|  | Summer | SKSK16 | CTGGCATA | GGTGCGAA |
|  |  | SKSK17 | CTGGCATA | GTCGTAGA |
|  |  | SKSK18 | CTGGCATA | TCTTCACA |
|  |  | SKSK19 | CTGGCATA | TTCACGCA |
|  |  | SKSK20 | CTGGCATA | AATCCGTC |
|  |  | SKSK21 | GCAAGCTA | CGACATAG |
|  |  | SKSK22 | GAGCAACT | AGACGTAC |
|  |  | SKSK23 | GCTCACAT | ACCGATCT |
|  |  | SKSK24 | CTACACGT | CTCATGAC |
|  |  | SKSK25 | TATACGCC | CCGAATAG |
|  |  | SKSK26 | GCCTAAGA | GTACCAGA |
|  |  | SKSK27 | CTTCCAGA | CTTCGAAC |
|  |  | SKSK28 | CGATCAAG | AACATGGC |
|  |  | SKSK29 | CCTAGCTA | CGATAACG |
|  |  | SKSK30 | ACGGACTA | GCTCAAGA |
|  | Autumn | SKSK31 | AAGCGTCA | ATACGGCA |
|  |  | SKSK32 | AGCCACTT | CTAGCGAA |
|  |  | SKSK33 | TCGCCTAA | CACTAAGG |
|  |  | SKSK34 | GGCACTAA | GCTTACAC |
|  |  | SKSK35 | GTAACAGC | ACGTACCT |
|  |  | SKSK36 | TAAGCAGC | ACGCCTAT |
|  |  | SKSK37 | CCGAGTAA | TCAACGGA |
|  |  | SKSK38 | GACAAGCT | ATGAGACC |
|  |  | SKSK39 | CTGTAACC | TAGGCAAC |
|  |  | SKSK40 | TAATCCGC | ATCAAGCG |
|  |  | SKSK41 | TAACTCGC | ATCATCCG |
|  |  | SKSK42 | GCCGTAAT | CCTGAGAA |
|  |  | SKSK43 | CAGCAATG | GTGCAACA |
|  |  | SKSK44 | CCTTACGA | ATACTGCC |
|  |  | SKSK45 | AGTCCGAA | TATACCGC |
|  | Winter | SKSK46 | CAATGCAG | GAACAGCT |
|  |  | SKSK47 | CTCTAAGC | CATCTAGC |
|  |  | SKSK48 | CATGACGA | TGAACAGC |
|  |  | SKSK49 | ATCGTACC | TACCTACG |
|  |  | SKSK50 | AACAGGTC | CGTTCACA |
|  |  | SKSK51 | AGCATCTC | GTGAACCA |
|  |  | SKSK52 | CAACTGAG | ACATGTCC |
|  |  | SKSK53 | GAATGACC | CATCCGAT |
|  |  | SKSK54 | CATTCGCA | CCTCATAG |
|  |  | SKSK55 | AGGTACCA | TTCGCAAC |
|  |  | SKSK56 | CAATGGCA | GCCTAACT |
|  |  | SKSK57 | TATACGCC | CCGAATAG |
|  |  | SKSK58 | AAGCGTCA | ATACGGCA |
|  |  | SKSK59 | AGCCACTT | CTAGCGAA |
|  |  | SKSK60 | CAGTGAAC | CAATTCGC |

**Table S5** List of the abbreviations for the taxa in the diet of *C. zillii* used in the subsequent figures, tables and the main text.

| Prey taxa category | Taxon | Abbreviation | Prey taxa category | Taxon | Abbreviation |
| --- | --- | --- | --- | --- | --- |
| Phytoplankton | Chlorophyta | CHLO | Zooplankton | Arthropoda | ARTH |
|  | Bacillariophyta | BACI |  | Rotifera | ROTI |
|  | Haptophyta | HAPT |  | Cnidaria | CNID |
| Protozoa | Ciliophora | CILI | Zoobenthos | Bryozoa | BRYO |
|  | Microsporidia | MICR |  | Annelida | ANNE |
|  | Cercozoa | CERC |  | Nematoda | NEMA |
|  | Apicomplexa | APIC |  | Gastrotricha | GAST |
|  | Picozoa | PICO |  | Nemertea | NEME |
|  | Euglenozoa | EUGL |  | Mollusca | MOLL |
|  | Imbricatea | IMBR |  | Platyhelminthes | PLAT |
|  | Heterolobosea | HETE |  | Tardigrada | TARD |
|  | Endomyxa | ENDO |  | Porifera | PORI |
|  | Tubulinea | TUBU | Detritus | Streptophyta | STRE |
|  | Perkinsozoa | PERK |  | Ascomycota | ASCO |
|  | Foraminifera | FORA |  | Oomycota | OOMY |
|  | Evosea | EVOS |  | Basidiomycota | BASI |
|  | Discosea | DISC |  | Cryptomycota | CRYP |
|  |  |  |  | Chytridiomycota | CHYT |
|  |  |  |  | Mucoromycota | MUCO |
|  |  |  |  | Blastocladiomycota | BLAS |
|  |  |  |  | Zoopagomycota | ZOOP |

**Table S6** Results of Detrended Correspondence Analysis (DCA) between diet data and environmental variables for *C. zillii* from five sampling sites and four seasons of SKSK

| Parameters of DCA | Type of grouping | | | | | | | |
| --- | --- | --- | --- | --- | --- | --- | --- | --- |
|  | habitat | | | | seasons of SKSK | | | |
| Axes | 1 | 2 | 3 | 4 | 1 | 2 | 3 | 4 |
| Eigenvalues | 0.71 | 0.44 | 0.29 | 0.089 | 0.58 | 0.38 | 0.20 | 0.11 |
| Lengths of gradient | 3.57 | 2.33 | 2.56 | 1.21 | 3.21 | 2.83 | 2.90 | 1.69 |
| Pseudo-canonical correlation | 0.61 | 0.44 | 0.60 | 0.30 | 0.64 | 0.39 | 0.54 | 0.50 |
| Explained variation (cumulative) (%) | 24.35 | 39.59 | 49.39 | 52.42 | 16.25 | 26.99 | 32.67 | 35.76 |

**Table S7** Statistics of stomach fullness and empty indices of *C. zillii* from five sampling sites and four seasons of SKSK.

| Type of grouping | Population | Degrees of stomach fullness | | | | | | Total Full | Total stomach | Fullness Index (%) | Empty Index (%) |
| --- | --- | --- | --- | --- | --- | --- | --- | --- | --- | --- | --- |
|  |  | 0 (Empty) | 1 | 2 | 3 | 4 | 5 |  |  |  |  |
| habitat | DJ | 18 | 12 | 9 | 5 | 5 | 2 | 33 | 51 | 64.71 | 35.29 |
|  | NDJ | 20 | 13 | 8 | 8 | 4 | 2 | 35 | 55 | 63.64 | 36.36 |
|  | QDH | 61 | 27 | 12 | 7 | 2 | 1 | 49 | 110 | 44.55 | 55.45 |
|  | SKSK | 38 | 10 | 6 | 2 | 4 | 3 | 25 | 63 | 39.68 | 60.32 |
|  | XJ | 32 | 16 | 8 | 6 | 3 | 3 | 36 | 68 | 52.94 | 47.06 |
|  | Total | 169 | 78 | 43 | 28 | 18 | 11 | 178 | 347 | 51.30 | 48.70 |
| seasons of SKSK | Spring | 17 | 54 | 23 | 12 | 26 | 6 | 121 | 138 | 87.68 | 12.32 |
|  | Summer | 82 | 37 | 23 | 25 | 14 | 9 | 108 | 190 | 56.84 | 43.16 |
|  | Autumn | 263 | 36 | 24 | 18 | 33 | 14 | 125 | 388 | 32.22 | 67.78 |
|  | Winter | 249 | 32 | 25 | 17 | 13 | 27 | 114 | 363 | 31.40 | 68.60 |
|  | Total | 611 | 159 | 95 | 72 | 86 | 56 | 468 | 1079 | 43.37 | 56.63 |

**Table S8** The statistics of effective tags and OTUs of *C. zillii* for diet analysis between habitat types and seasons

| Items | Habitat type | | | | | Seasons of SKSK | | | |
| --- | --- | --- | --- | --- | --- | --- | --- | --- | --- |
|  | River | | Reservoir | | | Spring | Summer | Autumn | Winter |
|  | DJ | NDJ | QDH | SKSK | XJ |  |  |  |  |
| Number of original tags  (Mean ± SD) | 116485±14217 | 139385±5397 | 101835±28065 | 137978±5600 | 133471±903 | 88283±2101 | 87470±9324 | 105158±8823 | 101465±11983 |
| Number of raw tags  (Mean ± SD) | 115462±14191 | 138425±5391 | 101521±28021 | 137291±5513 | 132530±845 | 87686±2183 | 86528±9056 | 104572±8791 | 100701±11834 |
| Number of clean tags  (Mean ± SD) | 113975±14049 | 136837±5276 | 100743±27830 | 136344±5449 | 130921±836 | 87144±2217 | 85756±8833 | 103064±8772 | 99821±11719 |
| Number of effective tags  (Mean ± SD) | 111041±13106 | 121157±9487 | 92286±28891 | 126359±6492 | 130330±981 | 81608±3644 | 79908±10250 | 99437±9739 | 92457±11476 |
| Average length of effective tags (nt)  (Mean ± SD) | 317±11 | 311±2 | 312±3 | 311±2 | 315±0 | 312±5 | 308±10 | 304±11 | 307±5 |
| Q20 (%)  (Mean ± SD) | 98.91±0.21 | 99.01±0.11 | 99.00±0.09 | 99.04±0.02 | 99.02±0.02 | 99.15±0.13 | 99.07±0.19 | 98.88±0.26 | 99.25±0.15 |
| Q30 (%)  (Mean ± SD) | 96.03±0.59 | 96.30±0.33 | 96.12±0.23 | 96.11±0.05 | 96.37±0.06 | 96.57±0.51 | 96.44±0.59 | 95.94±0.76 | 96.91±0.55 |
| Percentage of effective tags (%)  (Mean ± SD) | 95.39±2.54 | 86.89±5.12 | 90.28±6.79 | 91.56±1.89 | 97.65±0.30 | 92.44±3.34 | 91.36±6.53 | 94.49±3.01 | 91.17±4.35 |
| Total OTUs  (Mean ± SD) | 475 | 475 | 475 | 475 | 475 | 2590 | 2590 | 2590 | 2590 |

Q20 and Q30 represent the percentage of bases with Phred value more than 20 (sequencing error rate less than 1%) and 30 (sequencing error rate less than 0.1%), respectively.

SD denotes standard deviation. OTUs represents Operational Taxonomic Units.

**Table S9** Weighted unifrac distances (lower triangular matrix) and Bray-Curtis distances (upper triangular matrix) of dietary differences between five sampling sites.

|  | DJ | NDJ | QDH | SKSK | XJ |
| --- | --- | --- | --- | --- | --- |
| DJ | - | 0.88^***^ | 0.99 | 0.99^***^ | 0.87 |
| NDJ | 0.60^***^ | - | 1.00^***^ | 1.00 | 0.84^***^ |
| QDH | 0.68 | 0.57^**^ | - | 0.96^***^ | 0.98 |
| SKSK | 0.88^**^ | 0.79^***^ | 0.56 | - | 1.00^***^ |
| XJ | 0.55^*^ | 0.45^***^ | 0.43 | 0.71 | - |

^*^ indicates pairwise distance was significant at α = 0.05. ^**^ indicates pairwise distance was significant at α = 0.01. ^***^ indicates pairwise distance was significant at α = 0.001.

**Table S10** Geographic distances (lower triangular matrix; km) and Schoener index values (upper triangular matrix) of dietary overlap between five sampling sites.

|  | DJ | NDJ | QDH | SKSK | XJ |
| --- | --- | --- | --- | --- | --- |
| DJ | - | 0.35 | 0.49 | 0.11 | 0.57 |
| NDJ | 581.29 | - | 0.37 | 0.11 | 0.37 |
| QDH | 841.68 | 1404.82 | - | 0.33 | 0.58 |
| SKSK | 533.61 | 1114.54 | 358.15 | - | 0.25 |
| XJ | 617.07 | 506.27 | 1211.33 | 1030.87 | - |

**Table S11** Weighted unifrac distances (lower triangular matrix) and Bray-Curtis distances (upper triangular matrix) of dietary differences between four seasons in SKSK.

|  | Spring | Summer | Autumn | Winter |
| --- | --- | --- | --- | --- |
| Spring | - | 0.86^***^ | 0.95^***^ | 0.89 |
| Summer | 0.56^**^ | - | 0.80^*^ | 0.77 |
| Autumn | 0.56 | 0.46^*^ | - | 0.75^***^ |
| Winter | 0.47^**^ | 0.32 | 0.39^**^ | - |

^*^ indicates pairwise distance was significant at α = 0.05. ^**^ indicates pairwise distance was significant at α = 0.01. ^***^ indicates pairwise distance was significant at α = 0.001.

**Table S12** Bray-Curtis distances (lower triangular matrix) and Schoener index values (upper triangular matrix) of dietary overlap between four seasons in SKSK.

|  | Spring | Summer | Autumn | Winter |
| --- | --- | --- | --- | --- |
| Spring | - | 0.50 | 0.50 | **0.64** |
| Summer | 0.86^***^ | - | **0.64** | **0.74** |
| Autumn | 0.95^***^ | 0.80^*^ | - | **0.61** |
| Winter | 0.89 | 0.77 | 0.75^***^ | - |

^*^ indicates pairwise distance was significant at α = 0.05. ^***^ indicates pairwise distance was significant at α = 0.001.

Schoener indices greater than 0.6 are shown in bold.

**Table S13** Optimal multivariable linear regression modeling of Shannon-Wiener index, Pielou’s evenness index and Niche breadth index in relation to environmental factors for *C. zillii* from 5 sampling sites

|  |  | Unstandardized Coefficients | | Standardized Coefficients |  |  | Full Model Statistics | | |
| --- | --- | --- | --- | --- | --- | --- | --- | --- | --- |
| Response variables | Explanatory variables | B | S.E. | Beta | *t* | *Pr* (>︱*t*︱) | Adjusted *R*^2^ | *F* | *Pr* (*>F*) |
| Shannon-Wiener index | TEMP | 0.55 | 0.13 | 0.81 | 4.17 | 0.001 | 0.46 | 7.27 | 0.002 |
|  | DO | 0.53 | 0.30 | 0.30 | 1.79 | 0.090 |  |  |  |
|  | COND | 0.011 | 0.003 | 0.84 | 4.17 | 0.001 |  |  |  |
| Pielou’s evenness index | TEMP | 0.080 | 0.017 | 0.84 | 4.65 | 0.000 | 0.54 | 9.75 | 0.000 |
|  | DO | 0.077 | 0.039 | 0.31 | 1.98 | 0.063 |  |  |  |
|  | COND | 0.002 | 0.000 | 0.92 | 4.99 | 0.000 |  |  |  |
| Niche breadth index | TEMP | -0.090 | 0.12 | -0.14 | -0.76 | 0.46 | 0.53 | 9.19 | 0.001 |
|  | DO | -0.19 | 0.27 | -0.11 | -0.70 | 0.49 |  |  |  |
|  | COND | 0.008 | 0.002 | 0.65 | 3.47 | 0.003 |  |  |  |

**Table S14**  Optimal multivariable linear regression modeling of Shannon-Wiener index, Pielou’s evenness index and Niche breadth index in relation to environmental factors of *C. zillii* from four seasons of SKSK.

|  |  | Unstandardized Coefficients | | Standardized Coefficients |  |  | Full Model Statistics | | |
| --- | --- | --- | --- | --- | --- | --- | --- | --- | --- |
| Response variables | Explanatory variables | B | S.E. | Beta | *t* | *Pr* (>︱*t*︱) | Adjusted *R*^2^ | *F* | *Pr* (*>F*) |
| Shannon-Wiener index | pH | -0.42 | 0.72 | -0.12 | -0.58 | 0.57 | -0.014 | 0.86 | 0.53 |
|  | COND | -0.004 | 0.009 | -0.093 | -0.47 | 0.64 |  |  |  |
|  | TD | -0.008 | 0.015 | -0.11 | -0.50 | 0.62 |  |  |  |
|  | PI | 0.037 | 0.84 | 0.010 | 0.043 | 0.97 |  |  |  |
|  | TP | 37.16 | 24.85 | 0.31 | 1.50 | 0.14 |  |  |  |
|  | TN | -0.054 | 0.84 | -0.012 | -0.064 | 0.95 |  |  |  |
| Pielou’s evenness index | pH | -0.025 | 0.078 | -0.063 | -0.33 | 0.75 | 0.048 | 1.50 | 0.20 |
|  | COND | 0.000 | 0.001 | -0.097 | -0.51 | 0.61 |  |  |  |
|  | TD | 0.000 | 0.002 | -0.042 | -0.19 | 0.85 |  |  |  |
|  | PI | -0.006 | 0.092 | -0.015 | -0.066 | 0.95 |  |  |  |
|  | TP | 5.42 | 2.69 | 0.40 | 2.012 | 0.049 |  |  |  |
|  | TN | -0.10 | 0.091 | -0.21 | -1.14 | 0.26 |  |  |  |
| Niche breadth index | pH | -1.34 | 1.10 | -0.25 | -1.21 | 0.23 | -0.055 | 0.49 | 0.81 |
|  | COND | 0.003 | 0.014 | 0.047 | 0.24 | 0.82 |  |  |  |
|  | TD | 0.007 | 0.023 | 0.075 | 0.32 | 0.75 |  |  |  |
|  | PI | 0.85 | 1.29 | 0.16 | 0.66 | 0.52 |  |  |  |
|  | TP | 12.18 | 38.032 | 0.067 | 0.32 | 0.75 |  |  |  |
|  | TN | 0.21 | 1.29 | 0.032 | 0.16 | 0.87 |  |  |  |
